# Supplementary material for: A Compartmental Mathematical Model to Assess the Impact of Vaccination, Isolation, and Key Epidemiological Parameters on Mpox Control
Source: Med Sci (Basel). 2025 Oct 10;13(4):226. doi: 10.3390/medsci13040226 (PMC12551027; doi:10.3390/medsci13040226)
Supplement: Supplementary file 1 [file medsci-13-00226-s001.zip › Supplementary Methods.pdf]

SUPPLEMENTARY METHODS

1.1. Simulations and sensitivity analysis of the model

1.1.1. Simulations

Given the reported variability in the average incubation period in times and ranges $(1/\eta)$ (Yuan et al., 2022; Brand et al., 2023; Yang et al., 2023; Zhang et al., 2024), we used a range of 5-21 days and an average of 7-9 days. Based on this interval, the incubation rate (η) was calculated as $\left[\frac{1}{21}, \frac{1}{5}\right] = [0.0476, 0.2]$. Similar considerations were applied to the infectious period $(1/\gamma)$, which ranges from 3 to 21 (or 28) days (Yang et al., 2023; Zhang et al., 2024) with an average of 6-11 days (Brand et al., 2023; Yang et al., 2023). This resulted in a range value for the recovery rate (γ) range value of $\left[\frac{1}{28}, \frac{1}{3}\right] = [0.0357, 0.3333]$. A median time of loss of immunity for vaccination $(1/\kappa)$ of 219 days was considered from a range of 49 to 315 days (Hazra et al., 2024). This translates to a range for the rate of loss of immunity from vaccination of $\left[\frac{1}{315}, \frac{1}{49}\right] = [0.00317, 0.0204]$. Similarly, the time for loss of natural immunity $(1/\sigma)$ was considered within the range of 25-364 days with a median value of 112 days (Hazra et al., 2024), resulting in a calculated rate of loss of natural immunity (σ) of $\left[\frac{1}{364}, \frac{1}{25}\right] = [0.0027, 0.04]$. Based on the average progression rate from isolated to recovered class (δ) of 0.52 $year^{-1}$ (Peter et al., 2022; Molla et al., 2023), we calculated this rate as 0.52/365 = 0.00142 day^{-1}. We further assumed an interval of $[0.00001, 0.01]$. The isolation rate (φ) was estimated considering the proportion identified as a suspected case of 2.0 $year^{-1}$ (Peter et al., 2022; Leandry & Mureithi, 2023) equivalent to 2/365 = 0.00548 day^{-1}, and the reported rate of identifying a suspected case of monkeypox 0.0081 day^{-1} (Ngungu et al., 2023). Therefore, we assumed an interval of $[0.00001, 0.01]$ that could encompass these values. An average vaccination rate of 0.02 (2% of the susceptible population vaccinated per day) was assumed. Additionally, we considered the lowest vaccination speed of 0.001 (0.1%) and the highest vaccination rate of 0.05 (5%).

The recruitment rate (Λ) depends on the specific characteristics of each study population. We estimated its value by multiplying the birth rate (in days) by the total population for a one-year period. We used the most recent data available: birth rate from 2022 and total population from 2021. For the birth rate, we considered a range based on reported values across different countries. The lower limit represents the lowest birth rate observed in any country (Hong Kong SAR, China) according to World Bank data from 2022 (World

Bank, 2022a). The upper limit reflects the highest reported rate (Niger). Considering a year of 365 days and multiplied this by the estimated population from the most recent year with available data (World Bank, 2022b): $\left[\frac{5}{1000x365} \times 7{,}346.10 \times 10^3, \frac{45}{1000x365} \times 26{,}207.98 \times 10^3\right] = [100.631507{,}3231.12082]$. Similarly, an interval for the natural death rate (μ) was established. We considered death rates reported in various countries, using the lowest value (Qatar) and highest value (Bulgaria) to define the range (World Bank, 2022c): $\left[\frac{1}{1000x365}, \frac{22}{1000x365}\right] = [2.73972603 \times 10^{-6}, 6.02739726 \times 10^{-5}]$. Finally, the lethality rate (ω) has been reported with various values. These range from 0.00008 (Ngungu et al., 2023) to 0.0003125 (Vogel, 2022) and 0.0005479 (0.2/365) (Leandry & Mureithi, 2023), up to a broader range of 0.03 – 0.11 (Vogel, 2022). Considering this variability, a range of values of $[0.00008, 0.11]$ will be used for further analysis. All these values are summarized in Table 1.

1.1.2. Global sensitivity of the model

In dynamical systems modeled by ordinary differential equations, sensitivity studies are crucial. These studies allow us to estimate the uncertainty of output variables as a function of the input factors. A global sensitivity analysis based on variance decomposition, like Sobol indices estimation, enables us to work across the entire parameter space, even for discontinuous functions. Global sensitivity methods quantify the uncertainty of model output factors based on a sampling of probability density functions assigned to the input factors. This approach allows us to explore the entire range of variation of input factors and vary all parameters simultaneously (López-Cruz et al., 2012; Balesdent et al., 2016).

Since a dynamic system can be expressed vectorially as (Balesdent et al., 2016; Sobol, 2001):

$$Y = f(X) = f(X_1, X_2, \dots, X_k)$$

where the mathematical model consists of k input factors $X = (X_1, X_2. \dots, X_k)^T$ and f is a black box function (arbitrary function) and where Y is the output of interest.

In contrast, variance decomposition methods posit that the variance of the output variable can be quantified as the sum of contributions from input variables, factors, and their interactions. Within this framework, Sobol (Sobol, 1990; Balesdent et al., 2016) showed the possibility of a single functional decomposition of f:

$$f(\mathrm{X}) = f_0 + \sum_{j=1}^{k} f_j(X_j) + \sum_{i<j}^{k} f_{ij}(X_i, X_j) + \cdots + f_{1\cdots k}(X_1, X_2, \dots, X_k)$$

where $f_0 = \mathbb{E}(f(X)) = \int_\Omega f(X) g(\mathrm{X}) d\mathrm{X}$. Ω is the k-dimensional cube $[0,1]^k$, and the inputs factors have a Uniform distribution $U_{[0,1]}$ that it is represented by the probability density function g. Moreover, each factor of the previous equation is defined by a relationship of the mathematical expectation of conditional probabilities

$$f_j(X_j) = \mathbb{E}(f(X) \mid X_j) - f_0$$

$$f_{ij}(X_i, X_j) = \mathbb{E}(f(\mathrm{X}) \mid X_i, X_j) - \mathbb{E}(f(\mathrm{X}) \mid X_i) - \mathbb{E}(f(\mathrm{X}) \mid X_j) + f_0$$

And $f_{1,\cdots,k}(X_1, X_2, \dots X_k)$ is defined as the difference between $f(X)$ and the sum of all functions of increasing dimension, such that equation (29) is verified. Furthermore, each decomposition function satisfies the orthogonality condition.

Building on this concept, Sobol (Sobol, 2001; Balesdent et al., 2016) introduced global sensitivity indices for nonlinear mathematical models. These indices are based on the decomposition of the output variable's variance, as shown here:

$$\mathbb{V}(Y) = \sum_{j=1}^{k} \mathbb{V}_j(Y) + \sum_{1 \le i < j}^{k} \mathbb{V}_{ij}(Y) + \cdots + \mathbb{V}_{123\dots k}(Y)$$

Where the total output variance is $\mathbb{V}(Y)$, also $\mathbb{V}_j(Y) = \mathbb{V}[\mathbb{E}(Y|X_j)]$, $\mathbb{V}_{ij}(Y) = \mathbb{V}[\mathbb{E}(Y|X_i, X_j)] - \mathbb{V}_i(Y) - \mathbb{V}_j(Y)$ and $\mathbb{V}_{123\dots k}(Y) = \mathbb{V}[\mathbb{E}(Y|X_1, X_2, \dots, X_k)] - \sum_{i=1}^{k} \mathbb{V}_i$ denote the variance of f_j, f_{ij}, and $f_{123\dots k}$, respectively. Moreover, the total variance can be written by $\mathbb{V}(Y) = \mathbb{V}[\mathbb{E}(Y|X_j)] + \mathbb{E}[\mathbb{V}(Y|X_j)]$ (Balesdent et al., 2016; López-Cruz et al., 2012).

Taking this into account, the normalized first order sensitivity index ($S_j = S1$) is defined by (Balesdent et al., 2016; López-Cruz et al., 2012):

$$S_j = \frac{\mathbb{V}[\mathbb{E}(Y|X_j)]}{\mathbb{V}(Y)} = \frac{\mathbb{V}_j(Y)}{\mathbb{V}(Y)}$$

This index measures the variance of the output variable Y respect to the input factor X_j, and is also called “main effect index” because it indicates the individual contribution of each parameter to the variance of the model output. A high value of S1 for a parameter indicates a significant influence on the model's output variability. In the context of our model, for instance, a high S1 for a parameter signifies a substantial impact on the symptomatic infected population (I).

Additionally, the normalized second order sensitivity index ($S_{ij} = S2$) is given by (Balesdent et al., 2016):

$$S_{ij} = \frac{\mathbb{V}[\mathbb{E}(Y|X_i, X_j)] - \mathbb{V}_i(Y) - \mathbb{V}_j(Y)}{\mathbb{V}(Y)} = \frac{\mathbb{V}_{ij}(Y)}{\mathbb{V}(Y)}$$

S_{ij} quantifies the interaction between X_i and X_j. S2 captures the joint effect of parameter pairs on the model's output variance. A high S2 value for a parameter pair indicates that their interaction significantly influences output variability. For example, imagine parameter A has a high S_1 and parameter B has a high S_2 specifically with A. This suggests that the interplay between A and B is crucial in explaining the variation of the infected population.

Finally, the total Sobol sensitivity index ($ST_j = ST$) related to X_j can be written by (Sobol, 2001; Balesdent et al., 2016; López-Cruz et al., 2012)

$$ST_j = S_j + \sum_{1 \leq i < j}^{k} S_{ij} + \cdots + S_{123\ldots k}$$

Alternatively, ST can be calculated as (López-Cruz et al., 2012; Sobol, 2001):

$$ST_j = 1 - \frac{\mathbb{V}[\mathbb{E}(Y|X_{\sim j})]}{\mathbb{V}(Y)} = 1 - \frac{\mathbb{V}_{\sim j}}{\mathbb{V}(Y)} = \frac{\mathbb{E}[\mathbb{V}(Y|X_{\sim j})]}{\mathbb{V}(Y)}$$

Where $X_{\sim j} = (X_1, X_2, \ldots, X_{j-1}, X_{j+1}, \ldots, X_k)$ indicates a vector that not consider the effect of the input X_j (Output Y variability for all input variables except X_j is analyzed by setting the input variable X_j to an $x_j$value: $\mathbb{V}(Y|X_j = x_j) = \mathbb{E}(Y^2|X_j = x_j) - \mathbb{E}(Y|X_j = x_j)^2$, in order to consider all possible x_j, the expectation of conditional variance is considered). The ST index captures the total contribution of a parameter to the output variance. This includes both the parameter's individual effect (S1) and its interaction effects with other parameters (S2 and/or higher order indices). A high ST value indicates a highly influential parameter on the model's output variability, either through its individual effect or its interactions with others.

Computationally, these indices can be calculated using methods like Monte Carlo simulations to estimate the expected values of the variances (López-Cruz et al., 2012). In summary, S1, S2 and ST all represent the importance and influence of model input parameters on the output variable's variability. Higher values of these indices indicate a stronger influence of the corresponding factors on the model's output. When the sum of all S_j or ST_j equals 1, the model is called additive, otherwise it is non-additive, and values equal to zero are interpreted as the model is not sensitive to that input factor; in general, $0 \leq S_j \leq ST_j \leq 1$ (López-Cruz et al., 2012; Sobol, 2001).

2. Analysis and mathematical validation of the proposed model's properties

2.1. Model building

2.1.1. Positivity

Theorem 1. Let the ordinary differential equation system *(1)* of the Mpox disease dynamics be given with initial conditions $S(0) \geq 0, E(0) \geq 0, I(0) \geq 0, A(0) \geq 0, Q(0) \geq 0, R(0) \geq 0, V(0) \geq 0$, then, system *(1)* has nonnegative solutions $(S, E, I, A, Q, R, V) \in \mathbb{R}_+^7$ for all time $t > 0$.

Proof. Consider system *(1)* with nonnegative initial conditions $S(0), E(0), I(0), A(0), Q(0), R(0), V(0) \geq 0$. To avoid the removable singularity when $N(t) = 0$ we define the incidence term by continuity: whenever $N(t) = 0$ we set $\frac{(\beta_1 I + \beta_2 A)S}{N} = 0$ (equivalently, the expression is continuously extended at $N = 0$ since then $S = I = A = 0$). The right-hand side of system *(1)* is locally Lipschitz on the nonnegative orthant with this interpretation, so solutions exist and are unique.

Let $x(t) = (S, E, I, A, Q, R, V)$. Suppose by contradiction that there exists a first time $t^* > 0$ and an index i such that $x_i(t^*) = 0$ and for some $\varepsilon > 0$ we have $x_i(t) < 0$ for $t \in (t^*, t^* + \varepsilon)$. By minimality of t^* we have $x_j(t) \geq 0$ for all j and all $t \in [0, t^*]$.

We check each coordinate at the boundary value $x_i = 0$:

- If $S(t^*) = 0$ then, since terms proportional us S vanish at $S = 0$,
$$S^{\cdot}(t^*) = \Lambda + \kappa V(t^*) + \sigma R(t^*) \geq 0.$$
- If $E(t^*) = 0$ then
$$E^{\cdot}(t^*) = \left.\frac{(\beta_1 I + \beta_2 A)S}{N}\right|_{t^*} \geq 0,$$

using the continuous extension at $N = 0$.

- If $I(t^*) = 0$ then $I^{\cdot}(t^*) = p\eta E(t^*) \geq 0$.
- If $A(t^*) = 0$ then $A^{\cdot}(t^*) = (1 - p)\eta E(t^*) \geq 0$.
- If $Q(t^*) = 0$ then $Q^{\cdot}(t^*) = \phi I(t^*) \geq 0$.
- If $R(t^*) = 0$ then $R^{\cdot}(t^*) = \gamma I(t^*) + \gamma A(t^*) + \delta Q(t^*) \geq 0$.
- If $V(t^*) = 0$ then $V^{\cdot}(t^*) = \varepsilon v S(t^*) \geq 0$.

In every case the right-hand side at the first hitting time is nonnegative, contradicting that the component becomes negative immediately after t^*. Thus, no component can cross to negative values, and all components remain nonnegative for all $t \geq 0$.

Finally, for the total population $N(t) = \sum x_i(t)$ observe that if at some time t^* one had $N(t^*) = 0$, then necessarily all compartments are zero at t^* and hence

$$\dot{N}(t^*) = \Lambda > 0,$$

so $N(t)$ immediately increases and cannot becone negative. Therefore $N(t) \geq 0$ for all $t \geq 0$.

This completes the positivity proof (we use continuous extension at $N = 0$ to remove the removable singularity in the incidence terms).■

2.1.2. Boundedness

Theorem 2. The invariant feasible region of the system *(1)* for the Mpox disease, is a non-negative region defined by

$$\Omega = \left\{ \big(S(t), E(t), I(t), A(t), Q(t), R(t), V(t)\big) \in \mathbb{R}_+^7 : N(t) \leq \frac{\Lambda}{\mu} \right\}$$

Where $N(t) = S(t) + E(t) + I(t) + A(t) + Q(t) + R(t) + V(t)$, and Ω attracts all the solutions in $\mathbb{R}_+^7$.

Proof. Since the relation between the whole variables was established in *(2)*, and adding all the equations of the system *(1)*, the total population change rate is given by:

$$\frac{dN}{dt} = \frac{dS}{dt} + \frac{dE}{dt} + \frac{dI}{dt} + \frac{dA}{dt} + \frac{dQ}{dt} + \frac{dR}{dt} + \frac{dV}{dt}$$

$$\frac{dN}{dt} = \Lambda - \mu S - \mu E - \mu I - \mu A - \mu Q - \mu R - \mu V - \omega I - \omega Q$$

$$\frac{dN}{dt} = \Lambda - \mu(S + E + I + A + Q + R + V) - \omega(I + Q)$$

$$\frac{dN}{dt} = \Lambda - \mu N - \omega(I + Q)$$

It is clear to see that

$$\frac{dN}{dt} \leq \Lambda - \mu N$$

$$\frac{dN}{dt} + \mu N \leq \Lambda$$

Solving the inequality by the integration factor method $\left(e^{\int_{t_0}^{t} \mu d\tau} = e^{\mu t}, t_0 = 0\right)$, then:

$$\frac{dN}{dt} e^{\mu t} + \mu e^{\mu t} N \leq \Lambda e^{\mu t}$$

$$\frac{d(Ne^{\mu t})}{dt} \leq \Lambda e^{\mu t}$$

$$\int_0^t \frac{d(Ne^{\mu \tau})}{d\tau} d\tau \leq \int_0^t \Lambda e^{\mu \tau} d\tau$$

$$N(t)e^{\mu t} - N(0)e^{\mu \cdot 0} \leq \frac{\Lambda}{\mu}e^{\mu t} - \frac{\Lambda}{\mu}e^{\mu \cdot 0}$$

$$N(t) \leq \frac{\Lambda}{\mu} + \left(N(0) - \frac{\Lambda}{\mu}\right)e^{-\mu t}$$

Taking the limit, $\lim_{t\to\infty} N(t) \leq \frac{\Lambda}{\mu}$, it means that when $t \to \infty$, then $N(t) \leq \frac{\Lambda}{\mu}$. Therefore, Ω is positively invariant and attracts all solution in $\mathbb{R}_{+}^{7}$. ■

2.1.3. Existence of the equilibrium points

2.1.3.1. Disease-Free Equilibrium Point

Theorem 3. System *(1)* always has a unique disease-free equilibrium point.

Proof. Since a disease-free equilibrium point means no infected individuals, we set: $E = 0$, $I = 0$, $A = 0$, from which it follows that $-\delta Q - \omega Q - \mu Q = 0$, then Q =0, in addition, $-\mu R - \sigma R = 0$, so $R = 0$, moreover, $\Lambda - \varepsilon \nu S + \kappa V - \mu S = 0$, we get

$$(\varepsilon\nu + \mu)S - \kappa V = \Lambda, \tag{1}$$

according to the seventh differential equation of the system *(1)*: $\varepsilon \nu S - \kappa V - \mu V = 0$, so we have

$$V = \left(\frac{\varepsilon\nu}{\kappa + \mu}\right)S, \tag{2}$$

Replacing equation *(4)* in *(3)*, we obtain:

$$S = S_0^* = \frac{\Lambda(\kappa + \mu)}{\mu(\varepsilon\nu + \kappa + \mu)}. \tag{3}$$

Substituting equation *(5)* into equation *(4)*, we get:

$$V = V_0^* = \frac{\varepsilon\nu\Lambda}{\mu(\varepsilon\nu + \kappa + \mu)}. \tag{4}$$

We conclude that the disease-free equilibrium point is:

$$P_0^* = (S_0^*, 0,0,0,0,0, V_0^*) = \left(\frac{\Lambda(\kappa + \mu)}{\mu(\varepsilon\nu + \kappa + \mu)}, 0,0,0,0,0, \frac{\varepsilon\nu\Lambda}{\mu(\varepsilon\nu + \kappa + \mu)}\right). \blacksquare \tag{5}$$

From these findings, we can calculate the value of the total population in the disease-free equilibrium, as $N_0^* = S_0^* + V_0^* = S_0^* + \left(\frac{\varepsilon\nu}{\kappa+\mu}\right)S_0^* = \left(1 + \frac{\varepsilon\nu}{\kappa+\mu}\right)S_0^*$, we find

$$\frac{S_0^*}{N_0^*} = \left(\frac{\kappa + \mu}{\varepsilon\nu + \kappa + \mu}\right). \tag{6}$$

2.1.3.2. Estimation of the basic reproduction number ($\mathcal{R}_0$)

To calculate ($\mathcal{R}_0$), it was employed the next-generation matrix method (Van den Driessche & Watmough, 2002; Diekmann et al., 2010). This method analyzes the dynamics of infected compartments, such as exposed, symptomatic, asymptomatic, and

isolated individuals. We identified the matrices related to new infection generation ($\mathcal{F}$), and the flows of infected individuals due to other causes ($\mathcal{V}$, where ($\mathcal{V}^+$) represents the input matrix and ($\mathcal{V}^-$) refers to the output matrix) (Diekmann et al., 2010).

$$\mathcal{F} = \mathcal{F}_{(E,I,A,Q)} = \begin{pmatrix} \frac{(\beta_1 I + \beta_2 A)S}{N} \\ 0 \\ 0 \\ 0 \end{pmatrix} \tag{7}$$

$$\mathcal{V}^+ = \mathcal{V}^+_{(E,I,A,Q)} = \begin{pmatrix} 0 \\ p\eta E \\ (1-p)\eta E \\ \varphi I \end{pmatrix}, \mathcal{V}^- = \mathcal{V}^-_{(E,I,A,Q)} = \begin{pmatrix} \eta E + \mu E \\ \varphi I + \gamma I + \omega I + \mu I \\ \gamma A + \mu A \\ \delta Q + \omega Q + \mu Q \end{pmatrix}$$

$$\mathcal{V} = \mathcal{V}_{(E,I,A,Q)} = \mathcal{V}^- - \mathcal{V}^+ = \begin{pmatrix} \eta E + \mu E \\ \varphi I + \gamma I + \omega I + \mu I - p\eta E \\ \gamma A + \mu A - (1-p)\eta E \\ \delta Q + \omega Q + \mu Q - \varphi I \end{pmatrix} \tag{8}$$

Since the system involves nonlinear differential equations, we linearize equations *(9)* and *(10)* around the disease-free equilibrium point (Van den Driessche & Watmough, 2002). The Jacobian matrices for this linearization are calculated as follows:

$$F = \left.\frac{\partial \mathcal{F}_i}{\partial x_i}\right|_{P_0^*} = \begin{pmatrix} \left.\frac{\partial \mathcal{F}_E}{\partial E}\right|_{P_0^*} & \left.\frac{\partial \mathcal{F}_E}{\partial I}\right|_{P_0^*} & \left.\frac{\partial \mathcal{F}_E}{\partial A}\right|_{P_0^*} & \left.\frac{\partial \mathcal{F}_E}{\partial Q}\right|_{P_0^*} \\ \left.\frac{\partial \mathcal{F}_I}{\partial E}\right|_{P_0^*} & \left.\frac{\partial \mathcal{F}_I}{\partial I}\right|_{P_0^*} & \left.\frac{\partial \mathcal{F}_I}{\partial A}\right|_{P_0^*} & \left.\frac{\partial \mathcal{F}_I}{\partial Q}\right|_{P_0^*} \\ \left.\frac{\partial \mathcal{F}_A}{\partial E}\right|_{P_0^*} & \left.\frac{\partial \mathcal{F}_A}{\partial I}\right|_{P_0^*} & \left.\frac{\partial \mathcal{F}_A}{\partial A}\right|_{P_0^*} & \left.\frac{\partial \mathcal{F}_A}{\partial Q}\right|_{P_0^*} \\ \left.\frac{\partial \mathcal{F}_Q}{\partial E}\right|_{P_0^*} & \left.\frac{\partial \mathcal{F}_Q}{\partial I}\right|_{P_0^*} & \left.\frac{\partial \mathcal{F}_Q}{\partial A}\right|_{P_0^*} & \left.\frac{\partial \mathcal{F}_Q}{\partial Q}\right|_{P_0^*} \end{pmatrix}$$

$$F = \begin{pmatrix} 0 & \frac{\beta_1 S_0^*}{N_0^*} & \frac{\beta_2 S_0^*}{N_0^*} & 0 \\ 0 & 0 & 0 & 0 \\ 0 & 0 & 0 & 0 \\ 0 & 0 & 0 & 0 \end{pmatrix} = \begin{pmatrix} 0 & \frac{\beta_1(\kappa+\mu)}{(\varepsilon\nu+\kappa+\mu)} & \frac{\beta_2(\kappa+\mu)}{(\varepsilon\nu+\kappa+\mu)} & 0 \\ 0 & 0 & 0 & 0 \\ 0 & 0 & 0 & 0 \\ 0 & 0 & 0 & 0 \end{pmatrix} \tag{9}$$

$$V = \left.\frac{\partial \mathcal{V}_i}{\partial x_i}\right|_{P_0^*} = \begin{pmatrix} \left.\frac{\partial \mathcal{F}_E}{\partial E}\right|_{P_0^*} & \left.\frac{\partial \mathcal{F}_E}{\partial I}\right|_{P_0^*} & \left.\frac{\partial \mathcal{F}_E}{\partial A}\right|_{P_0^*} & \left.\frac{\partial \mathcal{F}_E}{\partial Q}\right|_{P_0^*} \\ \left.\frac{\partial \mathcal{F}_I}{\partial E}\right|_{P_0^*} & \left.\frac{\partial \mathcal{F}_I}{\partial I}\right|_{P_0^*} & \left.\frac{\partial \mathcal{F}_I}{\partial A}\right|_{P_0^*} & \left.\frac{\partial \mathcal{F}_I}{\partial Q}\right|_{P_0^*} \\ \left.\frac{\partial \mathcal{F}_A}{\partial E}\right|_{P_0^*} & \left.\frac{\partial \mathcal{F}_A}{\partial I}\right|_{P_0^*} & \left.\frac{\partial \mathcal{F}_A}{\partial A}\right|_{P_0^*} & \left.\frac{\partial \mathcal{F}_A}{\partial Q}\right|_{P_0^*} \\ \left.\frac{\partial \mathcal{F}_Q}{\partial E}\right|_{P_0^*} & \left.\frac{\partial \mathcal{F}_Q}{\partial I}\right|_{P_0^*} & \left.\frac{\partial \mathcal{F}_Q}{\partial A}\right|_{P_0^*} & \left.\frac{\partial \mathcal{F}_Q}{\partial Q}\right|_{P_0^*} \end{pmatrix}$$

$$V=\begin{pmatrix}\eta+\mu & 0 & 0 & 0\\ -p\eta & \varphi+\gamma+\omega+\mu & 0 & 0\\ -(1-p)\eta & 0 & \gamma+\mu & 0\\ 0 & -\varphi & 0 & \delta+\omega+\mu\end{pmatrix} \qquad (10)$$

the inverse of *(12)* must also be calculated:

$$\mathrm{V}^{-1}=\begin{pmatrix}\frac{1}{\eta+\mu} & 0 & 0 & 0\\ \frac{p\eta}{(\eta+\mu)(\varphi+\gamma+\omega+\mu)} & \frac{1}{\varphi+\gamma+\omega+\mu} & 0 & 0\\ \frac{(1-p)\eta}{(\eta+\mu)(\gamma+\mu)} & 0 & \frac{1}{\gamma+\mu} & 0\\ \frac{\varphi p\eta}{(\eta+\mu)(\delta+\omega+\mu)(\varphi+\gamma+\omega+\mu)} & \frac{\varphi}{(\varphi+\gamma+\omega+\mu)(\delta+\omega+\mu)} & 0 & \frac{1}{\delta+\omega+\mu}\end{pmatrix} \qquad (11)$$

The next generation matrix is then calculated using equations *(11)* and *(13)*. This matrix is defined as:

$$K=FV^{-1}$$

Where K is the following matrix:

$$\begin{pmatrix}\frac{\beta_1(\kappa+\mu)p\eta}{(\varepsilon\nu+\kappa+\mu)(\eta+\mu)(\varphi+\gamma+\omega+\mu)}+\frac{\beta_2(\kappa+\mu)(1-p)\eta}{(\varepsilon\nu+\kappa+\mu)(\eta+\mu)(\gamma+\mu)} & \frac{\beta_1(\kappa+\mu)}{(\varepsilon\nu+\kappa+\mu)(\varphi+\gamma+\omega+\mu)} & \frac{\beta_2(\kappa+\mu)}{(\varepsilon\nu+\kappa+\mu)(\gamma+\mu)} & 0\\ 0 & 0 & 0 & 0\\ 0 & 0 & 0 & 0\\ 0 & 0 & 0 & 0\end{pmatrix}$$

Using the next generation matrix methodology, the basic reproduction number ($\mathcal{R}_0$) is determined by the spectral radius of the next generation matrix. In simpler terms, it represents the maximum absolute value of the matrix's eigenvalues. In our case, the basic reproduction number is:

$$\mathcal{R}_0=\frac{(\kappa+\mu)\eta}{(\eta+\mu)(\varepsilon\nu+\kappa+\mu)}\left[\frac{p\beta_1}{(\varphi+\gamma+\omega+\mu)}+\frac{(1-p)\beta_2}{(\gamma+\mu)}\right] \qquad (12)$$

2.1.3.3. Endemic Equilibrium Point

Theorem 4. The system *(1)* admits a unique endemic equilibrium P^* with $I^*>0$ if and only if $\mathcal{R}_0>1$.

Proof. From the equilibrium equations we express all compartments linearly in terms of I :

$$E=a_E I, A=a_A I, Q=a_Q I, R=a_R I, \qquad (15)$$

where

$$a_E=\frac{\phi+\gamma+\omega+\mu}{p\eta}, a_A=\frac{(1-p)(\phi+\gamma+\omega+\mu)}{p(\gamma+\mu)}, a_Q=\frac{\phi}{\delta+\omega+\mu},$$

and

$$a_R=\frac{1}{\sigma+\mu}\left(\gamma+\frac{\gamma(1-p)(\phi+\gamma+\omega+\mu)}{p(\gamma+\mu)}+\frac{\delta\phi}{\delta+\omega+\mu}\right).$$

From the V-equation *(6)* and the first equation one finds

$$S = \frac{(\Lambda - \Pi I)(\kappa + \mu)}{\mu(\varepsilon\nu + \kappa + \mu)} = \alpha - \beta I, \tag{16}$$

with

$$\alpha = \frac{\Lambda(\kappa + \mu)}{\mu(\varepsilon\nu + \kappa + \mu)}, \beta = \frac{\Pi(\kappa + \mu)}{\mu(\varepsilon\nu + \kappa + \mu)},$$

and

$$\Pi = \frac{(\eta + \mu)(\phi + \gamma + \omega + \mu)}{p\eta} + \frac{\sigma}{\sigma + \mu}\left(\gamma + \frac{\gamma(1 - p)(\phi + \gamma + \omega + \mu)}{p(\gamma + \mu)} + \frac{\delta\phi}{\delta + \omega + \mu}\right).$$

The total population at equilibrium can be written as

$$N = S + E + I + A + Q + R + V = \frac{a_0}{\kappa + \mu}S + \Gamma I, \tag{17}$$

where $a_0 = \varepsilon\nu + \kappa + \mu$ and $\Gamma = a_E + 1 + a_A + a_Q + a_R$. Substituting $S = \alpha - \beta I$ gives linear relation

$$N = \frac{\Lambda}{\mu} + \left(\Gamma - \frac{\Pi}{\mu}\right)I. \tag{18}$$

The infectious-class balance at equilibrium yields (after algebraic manipulation using the definition of $\mathcal{R}_0$)

$$I(\Pi(\mathcal{R}_0 - 1) + \mu\Gamma) = \Lambda(\mathcal{R}_0 - 1). \tag{19}$$

Hence, for $R_0 \neq 1$,

$$I^* = \frac{\Lambda(\mathcal{R}_0 - 1)}{\Pi(\mathcal{R}_0 - 1) + \mu\Gamma}. \tag{20}$$

Since $\Pi > 0$ and $\Gamma \geq 0$, the denominator is positive for $\mathcal{R}_0 > 1$, whence $I^* > 0$ if and only if $\mathcal{R}_0 > 1$. The expression shows uniqueness because the linear equation in I admits a single solution. For $\mathcal{R}_0 = 1$ the unique equilibrium is the DFE with $I^* = 0$

From the seventh equation of the system *(1)*, we have

$$V = \left(\frac{\varepsilon\nu}{\kappa + \mu}\right)S \tag{21}$$

Similarly, from the second equation of the system *(1)*:

$$E = \left(\frac{\beta_1 I + \beta_2 A}{\eta + \mu}\right)\frac{S}{N} \tag{22}$$

And considering that we can rewrite the third and fourth equation of the system *(1)* as follows:

$$I = \left(\frac{p\eta}{\varphi + \gamma + \omega + \mu}\right)E, \qquad A = \left[\frac{(1 - p)\eta}{\gamma + \mu}\right]E$$

We replace those expressions into (22) and simplifying, we obtained:

$$1 = \left[\frac{\beta_1 p}{\varphi + \gamma + \omega + \mu} + \frac{(1 - p)\beta_2}{\gamma + \mu}\right]\frac{\eta S}{(\eta + \mu)N}$$

Also considering the $\mathcal{R}_0$ definition, we could rewrite the equation *(14)* in terms of $\mathcal{R}_0$, that is

$$1 = \frac{\mathcal{R}_0(\varepsilon\nu + \kappa + \mu)}{(\kappa + \mu)} \cdot \frac{S}{N}$$

So, we found that

$$S = \left(\frac{\kappa + \mu}{\varepsilon\nu + \kappa + \mu}\right) \cdot \frac{N}{\mathcal{R}_0} \tag{23}$$

From the expressions *(15)*, *(20)*, *(21)* and *(23)*, it is possible to assert that there is only one endemic equilibrium point of the form:

$$P^* = (S^*, E^*, I^*, A^*, Q^*, R^*, V^*) \tag{24}$$

With the following relations

$$S^* = \left(\frac{\kappa + \mu}{\varepsilon\nu + \kappa + \mu}\right) \cdot \frac{N^*}{\mathcal{R}_0}, \quad E^* = \left(\frac{\varphi + \gamma + \omega + \mu}{p\eta}\right) I^*, \quad I^* = \frac{\Lambda(\mathcal{R}_0 - 1)}{\Pi(\mathcal{R}_0 - 1) + \mu\Gamma},$$

$$A^* = \left[\frac{(1-p)(\varphi + \gamma + \omega + \mu)}{p(\gamma + \mu)}\right] I^*, \quad Q^* = \left(\frac{\varphi}{\delta + \omega + \mu}\right) I^*,$$

$$R^* = \left(\frac{1}{\sigma + \mu}\right)\left[\gamma + \frac{\gamma(1-p)(\varphi + \gamma + \omega + \mu)}{p(\gamma + \mu)} + \frac{\delta\varphi}{\delta + \omega + \mu}\right] I^*, \quad V^* = \left(\frac{\varepsilon\nu}{\kappa + \mu}\right) S^*$$

whose existence is conditional on that $\mathcal{R}_0 > 1$, where $N^* = S^* + E^* + I^* + A^* + Q^* + R^* + V^* = \frac{a_0}{\kappa+\mu} S^* + \Gamma I^*$, $\Pi = \left\{\frac{(\eta+\mu)(\varphi+\gamma+\omega+\mu)}{p\eta} + \left(\frac{\sigma}{\sigma+\mu}\right)\left[\gamma + \frac{\gamma(1-p)(\varphi+\gamma+\omega+\mu)}{p(\gamma+\mu)} + \frac{\delta\varphi}{\delta+\omega+\mu}\right]\right\}$, and $\Gamma = a_E + 1 + a_A + a_Q + a_R$, where $a_0 = \varepsilon\nu + \kappa + \mu$, $a_E = \frac{\phi+\gamma+\omega+\mu}{p\eta}$, $a_A = \frac{(1-p)(\phi+\gamma+\omega+\mu)}{p(\gamma+\mu)}$, $a_Q = \frac{\phi}{\delta+\omega+\mu}$, $a_R = \frac{1}{\sigma+\mu}\left(\gamma + \frac{\gamma(1-p)(\phi+\gamma+\omega+\mu)}{p(\gamma+\mu)} + \frac{\delta\phi}{\delta+\omega+\mu}\right)$.

This completes the proof of existence and uniqueness of the endemic equilibrium if only if $\mathcal{R}_0 > 1$. ■

2.1.4. Local stability analysis

2.1.4.1. Disease-Free Equilibrium Point

Theorem 5. The disease-free equilibrium point is locally asymptotically stable if and only if $\mathcal{R}_0 < 1$. Otherwise, it is unstable.

Proof. We evaluate the Jacobian matrix at the disease-free equilibrium point given in *(7)* and substitute the expression for $\mathcal{R}_0$ from *(14)*. This yields:

$$J(P_0^*) = \begin{pmatrix} -\varepsilon\nu - \mu & 0 & -\beta_1\left(\frac{\kappa+\mu}{\varepsilon\nu+\kappa+\mu}\right) & -\beta_2\left(\frac{\kappa+\mu}{\varepsilon\nu+\kappa+\mu}\right) & 0 & \sigma & \kappa \\ 0 & -(\eta+\mu) & \beta_1\left(\frac{\kappa+\mu}{\varepsilon\nu+\kappa+\mu}\right) & \beta_1\left(\frac{\kappa+\mu}{\varepsilon\nu+\kappa+\mu}\right) & 0 & 0 & 0 \\ 0 & p\eta & -(\varphi+\gamma+\omega+\mu) & 0 & 0 & 0 & 0 \\ 0 & (1-p)\eta & 0 & -(\gamma+\mu) & 0 & 0 & 0 \\ 0 & 0 & \varphi & 0 & -(\delta+\omega+\mu) & 0 & 0 \\ 0 & 0 & \gamma & \gamma & \delta & -(\sigma+\mu) & 0 \\ \varepsilon\nu & 0 & 0 & 0 & 0 & 0 & -(\kappa+\mu) \end{pmatrix}$$

Which characteristic equation can be written as follows:

$$P(\lambda) = \det(J(E_0^*) - \lambda I) = 0$$

In the above equation, λ represents the eigenvalue associated with the Jacobian matrix, and I denotes the identity matrix. Specifically, in our case:

$$(\lambda+\mu)(\lambda+\sigma+\mu)(\lambda+\varepsilon\nu+\kappa+\mu)(\lambda+\delta+\omega+\mu)(a_0\lambda^3+a_1\lambda^2+a_2\lambda+a_3)=0$$

Where

$$a_0=\varepsilon\nu+\kappa+\mu$$

$$a_1=(\varepsilon\nu+\kappa+\mu)(2\gamma+\eta+\varphi+\omega+3\mu)=a_0(2\gamma+\eta+\varphi+\omega+3\mu)$$

$$a_2=(\varepsilon\nu+\kappa+\mu)[(\eta+\mu)(\varphi+\gamma+\omega+\mu)+(\gamma+\mu)(\varphi+\gamma+\omega+\mu)+(\eta+\mu)(\gamma+\mu)] - \eta(\kappa+\mu)(p\beta_1+(1-p)\beta_2)$$

This term can be rewritten as a function of a_0 and $\mathcal{R}_0$ as follows:

$$a_2=a_0[(\eta+\mu)(\varphi+\gamma+\omega+\mu)+(\gamma+\mu)(\varphi+\gamma+\omega+\mu)+(\eta+\mu)(\gamma+\mu)] - \eta(\kappa+\mu)(p\beta_1+(1-p)\beta_2)$$

$$a_2=a_0(\gamma+\mu)(\eta+\varphi+\gamma+\omega+2\mu)+(\varepsilon\nu+\kappa+\mu)(\eta+\mu)(\varphi+\gamma+\omega+\mu) - \eta(\kappa+\mu)(p\beta_1+(1-p)\beta_2)$$

$$a_2=a_0(\gamma+\mu)(\varphi+\gamma+\omega+\mu)+\frac{\eta(\kappa+\mu)(\gamma+\mu)p\beta_1}{\varphi+\gamma+\omega+\mu}+\frac{\eta(\kappa+\mu)(\varphi+\gamma+\omega+\mu)(1-p)\beta_2}{\gamma+\mu}+a_0(\eta+\mu)(\varphi+2\gamma+\omega+2\mu)(1-\mathcal{R}_0)$$

And

$$a_3=(\gamma+\mu)(\varphi+\gamma+\omega+\mu)(\varepsilon\nu+\kappa+\mu)(\eta+\mu) - \eta(\kappa+\mu)[p\beta_1(\gamma+\mu)+(1-p)\beta_2(\varphi+\gamma+\omega+\mu)]$$

In terms of a_0 and $\mathcal{R}_0$:

$$a_3=(\gamma+\mu)(\varphi+\gamma+\omega+\mu)(\varepsilon\nu+\kappa+\mu)(\eta+\mu)(1-\mathcal{R}_0)$$

$$a_3=a_0(\gamma+\mu)(\varphi+\gamma+\omega+\mu)(\eta+\mu)(1-\mathcal{R}_0)$$

We can easily verify that the eigenvalues of the first four factors are negative:

$$\lambda_1=-\mu<0$$

$$\lambda_2=-(\sigma+\mu)<0$$

$$\lambda_3=-(\varepsilon\nu+\kappa+\mu)<0$$

$$\lambda_4=-(\delta+\omega+\mu)<0$$

The last three eigenvalues depend of the roots of the polynomial $a_0\lambda^3+a_1\lambda^2+a_2\lambda+a_3$, using the Routh–Hurwitz-Liénard–Chipart criteria (Wiggers & Pedersen, 2018) to ensure that the eigenvalues have a negative real part, the following relations must be satisfied:

$$a_i>0, i=0,1,2,3 \qquad \wedge \qquad a_1a_2-a_0a_3>0$$

it is possible to verify by simple inspection that $a_0>0, a_1>0$, in addition that $a_3>0$ implies $\mathcal{R}_0<1$ and therefore $a_2>0$. On the other hand, another condition implies that $a_1a_2>a_0a_3$, and it is satisfied in terms of the parameters:

$$a_0(2\gamma+\eta+\varphi+\omega+3\mu)\Big\{a_0(\gamma+\mu)(\varphi+\gamma+\omega+\mu)+\frac{\eta(\kappa+\mu)(\gamma+\mu)p\beta_1}{\varphi+\gamma+\omega+\mu}$$
$$+\frac{\eta(\kappa+\mu)(\varphi+\gamma+\omega+\mu)(1-p)\beta_2}{\gamma+\mu}$$
$$+a_0(\eta+\mu)(\varphi+2\gamma+\omega+2\mu)(1-\mathcal{R}_0)\Big\}$$
$$>a_0a_0(\gamma+\mu)(\varphi+\gamma+\omega+\mu)(\eta+\mu)(1-\mathcal{R}_0)$$

And

$$A_0+a_0(\gamma+\mu)(\varphi+\gamma+\omega+\mu)(\eta+\mu)(1-\mathcal{R}_0)$$
$$>a_0(\gamma+\mu)(\varphi+\gamma+\omega+\mu)(\eta+\mu)(1-\mathcal{R}_0)$$

Where

$$A_0=(2\gamma+\eta+\varphi+\omega+3\mu)\Big\{a_0(\gamma+\mu)(\varphi+\gamma+\omega+\mu)+\frac{\eta(\kappa+\mu)(\gamma+\mu)p\beta_1}{\varphi+\gamma+\omega+\mu}$$
$$+\frac{\eta(\kappa+\mu)(\varphi+\gamma+\omega+\mu)(1-p)\beta_2}{\gamma+\mu}+a_0(\eta+\mu)(\gamma+\mu)(1-\mathcal{R}_0)\Big\}$$
$$+a_0(\eta+\mu)(\varphi+\gamma+\omega+\mu)(1-\mathcal{R}_0)(\gamma+\eta+\varphi+\omega+2\mu)$$

Evaluating the Jacobian at the DFE we obtain the factorization of the characteristic polynomial

$$P(\lambda)=(\lambda+\mu)(\lambda+\sigma+\mu)(\lambda+\varepsilon\nu+\kappa+\mu)(\lambda+\delta+\omega+\mu)(a_0\lambda^3+a_1\lambda^2+a_2\lambda+a_3).$$

The first four linear factors give four negative eigenvalues. The remaining eigenvalues are roots of the cubic $p(\lambda)=a_0\lambda^3+a_1\lambda^2+a_2\lambda+a_3$ (with $a_0>0$). One checks that

$$a_3=a_0(\gamma+\mu)(\phi+\gamma+\omega+\mu)(\eta+\mu)(1-\mathcal{R}_0).$$

- If $\mathcal{R}_0<1$ then $a_3>0$ and, together with $a_0,a_1,a_2>0$ and the Routh-Hurwitz-Liénard-Chipart conditions (see text), all roots of the cubic have negative real parts; hence the DFE is locally asymptotically stable.
- If $\mathcal{R}_0>1$ then $a_3<0$. Notice that $p(0)=a_3<0$ while $p(\lambda)\to+\infty$ as $\lambda\to+\infty$. By the intermediate value theorem there exists $\lambda>0$ with $p(\lambda)=0$; therefore, the cubic has a positive real root, and the DFE has an eigenvalue with positive real part and is unstable."

Combining both directions gives the stated equivalence: the DFE is locally asymptotically stable if and only if $\mathcal{R}_0<1$.■

2.1.4.2. Endemic Equilibrium Point

Theorem 6. The endemic equilibrium point is locally asymptotically stable if and only if both $\mathcal{R}_0>1$ and conditions *(25)* are satisfied.

Proof.

Evaluating the Jacobian matrix at the endemic equilibrium point *(24)*, we get:

$$J(P^*) = \begin{pmatrix} -\frac{(\beta_1 I^* + \beta_2 A^*)}{N^*} - \varepsilon\nu - \mu & 0 & -\beta_1\frac{S^*}{N^*} & -\beta_2\frac{S^*}{N^*} & 0 & \sigma & \kappa \\ \frac{\beta_1 I^* + \beta_2 A^*}{N^*} & -(\eta+\mu) & \beta_1\frac{S^*}{N^*} & \beta_1\frac{S^*}{N^*} & 0 & 0 & 0 \\ 0 & p\eta & -(\varphi+\gamma+\omega+\mu) & 0 & 0 & 0 & 0 \\ 0 & (1-p)\eta & 0 & -(\gamma+\mu) & 0 & 0 & 0 \\ 0 & 0 & \varphi & 0 & -(\delta+\omega+\mu) & 0 & 0 \\ 0 & 0 & \gamma & \gamma & \delta & -(\sigma+\mu) & 0 \\ \varepsilon\nu & 0 & 0 & 0 & 0 & 0 & -(\kappa+\mu) \end{pmatrix}$$

Which characteristic equation can be written as follows:

$$b_0\lambda^7 + b_1\lambda^6 + b_2\lambda^5 + b_3\lambda^4 + b_4\lambda^3 + b_5\lambda^2 + b_6\lambda + b_7 = 0$$

Which coefficients can be seen in the Supplementary Material 1, to stablish the stability of the system according to the Routh–Hurwitz-Liénard–Chipart criteria (Wiggers & Pedersen, 2018) to ensure that the eigenvalues have a negative real part, the following relations must be satisfied:

$$\begin{gathered} b_i > 0, i = 0, \dots, 7; B_1 > 0; \\ B_1(B_3 + B_4) - B_2^2 > 0 \; and \\ B_1\left(2B_3B_6 + B_4B_6 - B_5^2\right) + B_2(B_3B_5 - B_2B_6) - B_3^2 > 0 \end{gathered} \qquad (25)$$

Where

$$B_1 = b_1b_2 - b_0b_3, \quad B_2 = b_1b_4 - b_0b_5, \quad B_3 = b_1b_6 - b_0b_7,$$
$$B_4 = b_3b_4 - b_2b_5, \quad B_5 = b_3b_6 - b_2b_7, \quad B_6 = b_5b_6 - b_4b_7,$$

To ensure these conditions are met, we analyzed the explicit term detailed in Supplementary Material 3. The positivity of coefficients b_i and B_1 depends on the following terms:

- The difference between the fraction of symptomatic infected individuals and the probability of developing symptoms, relative to the susceptible proportion:

  $$i - ps = \frac{I^*}{N^*} - \frac{pS^*}{N^*} = \frac{1}{N^*}(I^* - pS^*)$$

  In order to get a positive value:

  $$I^* - pS^* > 0$$

  Reordering and replacing it with identity *(23)* in the endemic equilibrium point:

  $$\frac{1}{p}\cdot I^* > \frac{(\kappa+\mu)}{(\varepsilon\nu+\kappa+\mu)}\cdot\frac{N^*}{\mathcal{R}_0}$$
  $$\Rightarrow \frac{1}{p}\cdot\frac{(\varepsilon\nu+\kappa+\mu)}{(\kappa+\mu)}\cdot\mathcal{R}_0 > \frac{N^*}{I^*} > 0$$

  In the last inequality as $0 < p \leq 1$, then $\frac{1}{p} \geq 1$, similarly $\frac{(\varepsilon\nu+\kappa+\mu)}{(\kappa+\mu)} \geq 1$, and $\frac{N^*}{I^*} > 1$, so to assure that this inequality is truth $\mathcal{R}_0$ must be satisfied: $\mathcal{R}_0 > 1$.

- The difference between the fraction of asymptomatic infected individuals and the probability of not developing symptoms, relative to the susceptible proportion, and replacing with $A = \left[\frac{(1-p)(\varphi+\gamma+\omega+\mu)}{p(\gamma+\mu)}\right] I$ (from A = a_AI, in equation 15):

$$a - (1-p)s = \frac{A^*}{N^*} - \frac{(1-p)S^*}{N^*} = \frac{(1-p)(\varphi+\gamma+\omega+\mu)}{p(\gamma+\mu)} \cdot \frac{I^*}{N^*} - \frac{(1-p)S^*}{N^*}$$

$$\Rightarrow a - (1-p)s = \frac{(1-p)}{N^*}\left(\frac{(\varphi+\gamma+\omega+\mu)}{p(\gamma+\mu)} \cdot I^* - S^*\right)$$

In order to get a positive value:

$$\frac{(\varphi+\gamma+\omega+\mu)}{p(\gamma+\mu)} \cdot I^* - S^* > 0$$

Reordering and replacing with identity *(23)* in the endemic equilibrium point:

$$\frac{(\varphi+\gamma+\omega+\mu)}{p(\gamma+\mu)} \cdot I^* > \frac{(\kappa+\mu)}{(\varepsilon\nu+\kappa+\mu)} \cdot \frac{N^*}{\mathcal{R}_0}$$

$$\Rightarrow \frac{1}{p} \cdot \frac{(\varphi+\gamma+\omega+\mu)}{(\gamma+\mu)} \cdot \frac{(\varepsilon\nu+\kappa+\mu)}{(\kappa+\mu)} \cdot \mathcal{R}_0 > \frac{N^*}{I^*} > 0$$

In the above inequality as $0 < p \le 1$ then $\frac{1}{p} \ge 1$, similarly $\frac{(\varepsilon\nu+\kappa+\mu)}{(\kappa+\mu)} \ge 1$, $\frac{(\varphi+\gamma+\omega+\mu)}{(\gamma+\mu)} \ge 1$ and $\frac{N^*}{I^*} > 1$, so to assure that this inequality is truth $\mathcal{R}_0$ must be satisfied: $\mathcal{R}_0 > 1$.

- Similarly, the following expressions:

$$\big((a-s)\varphi - \varepsilon\nu s\big)\delta - \varphi\varepsilon\nu s = a\delta\varphi - (\delta\varphi + \delta\varepsilon\nu + \varphi\varepsilon\nu)s$$

$$\big((a-s)\varphi - \varepsilon\nu s\big)\delta - \varphi\varepsilon\nu s = \frac{A^*}{N^*}\delta\varphi - (\delta\varphi + \delta\varepsilon\nu + \varphi\varepsilon\nu)\frac{S^*}{N^*}$$

$$\big((a-s)\varphi - \varepsilon\nu s\big)\delta - \varphi\varepsilon\nu s = \frac{1}{N^*}[A^*\delta\varphi - (\delta\varphi + \delta\varepsilon\nu + \varphi\varepsilon\nu)S^*]$$

In order to get a positive value:

$$A^*\delta\varphi - (\delta\varphi + \delta\varepsilon\nu + \varphi\varepsilon\nu)S^* > 0$$

Reordering and replacing with identity *(23)* in the endemic equilibrium point:

$$A^*\delta\varphi > (\delta\varphi + \delta\varepsilon\nu + \varphi\varepsilon\nu) \cdot \frac{(\kappa+\mu)}{(\varepsilon\nu+\kappa+\mu)} \cdot \frac{N^*}{\mathcal{R}_0}$$

$$\Rightarrow \frac{(\varepsilon\nu+\kappa+\mu)}{(\kappa+\mu)} \cdot \mathcal{R}_0 > \frac{(\delta\varphi + \delta\varepsilon\nu + \varphi\varepsilon\nu)}{\delta\varphi} \cdot \frac{N^*}{A^*} > 0$$

In this inequality is easy that $\frac{(\varepsilon\nu+\kappa+\mu)}{(\kappa+\mu)} \ge 1, \frac{(\delta\varphi+\delta\varepsilon\nu+\varphi\varepsilon\nu)}{\delta\varphi} \ge 1$, and $\frac{N^*}{A^*} \ge 1$, then $\mathcal{R}_0 > 1$.

- Finally, we found that

$$(a-s)\kappa-\varepsilon v s=ak-(\kappa+\varepsilon v)s=\frac{A^*}{N^*}\kappa-(\kappa+\varepsilon v)\frac{S^*}{N^*}=\frac{1}{N^*}[\kappa A^*-(\kappa+\varepsilon v)S^*]$$

In order to get a positive value:

$$\kappa A^*-(\kappa+\varepsilon v)S^*>0$$

Reordering and replacing with identity *(22)* in the endemic equilibrium point:

$$\kappa A^*>(\kappa+\varepsilon v)\cdot\frac{(\kappa+\mu)}{(\varepsilon v+\kappa+\mu)}\cdot\frac{N^*}{\mathcal{R}_0}$$

$$\Rightarrow\frac{(\varepsilon v+\kappa+\mu)}{(\kappa+\mu)}\cdot\mathcal{R}_0>\frac{(\kappa+\varepsilon v)}{\kappa}\cdot\frac{N^*}{A^*}>0$$

In this inequality is easy that $\frac{(\varepsilon v+\kappa+\mu)}{(\kappa+\mu)}\geq 1,\frac{(\kappa+\varepsilon v)}{\kappa}\geq 1,$ and $\frac{N^*}{A^*}\geq 1$, then $\mathcal{R}_0>1$.

As we noticed, when $\mathcal{R}_0>1$ we can assure that all above conditions ($b_i>0$ and $B_1>0$) are satisfied, and for the Theorem 4: A^*, I^*, S^*, and N^* are positive. We can conclude that if $\mathcal{R}_0>1$ the endemic equilibrium point is locally asymptotically stable. ■
