# Supplementary material for: A Compartmental Mathematical Model to Assess the Impact of Vaccination, Isolation, and Key Epidemiological Parameters on Mpox Control
Source: Med Sci (Basel). 2025 Oct 10;13(4):226. doi: 10.3390/medsci13040226 (PMC12551027; doi:10.3390/medsci13040226)
Supplement: Supplementary file 1 [file medsci-13-00226-s001.zip › Supplementary Figures.pdf]

SUPPLEMENTARY FIGURES

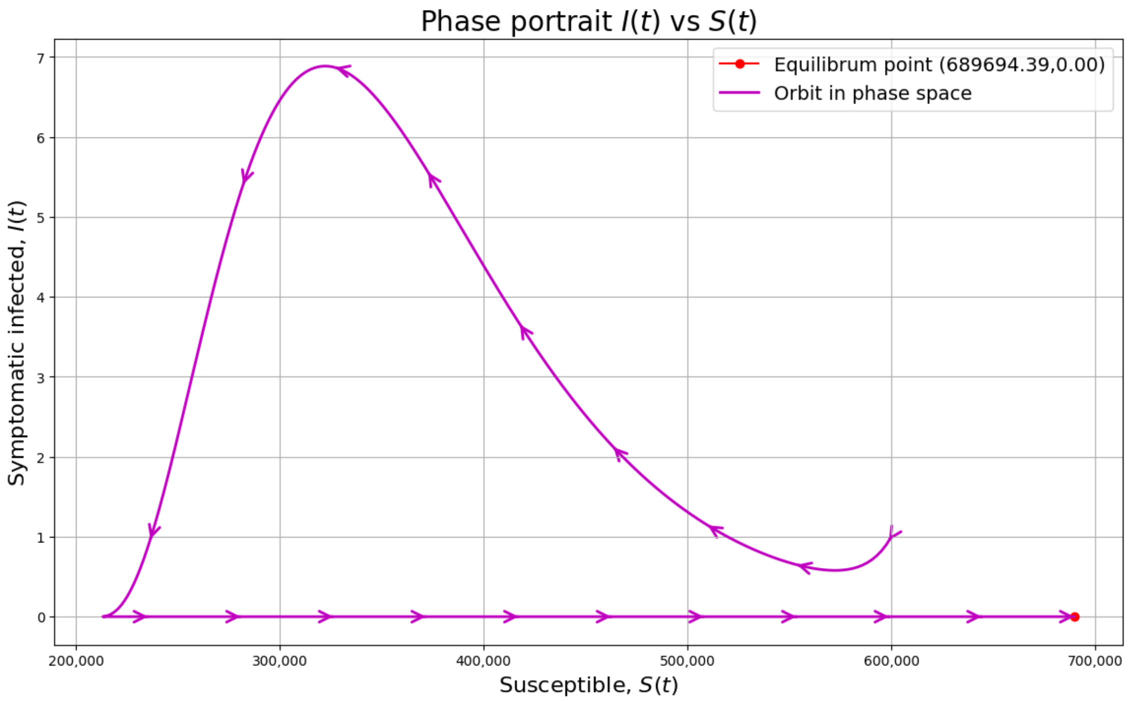

Figure S1. Phase portrait under a disease-free scenario, with R_0 = 0.6034.

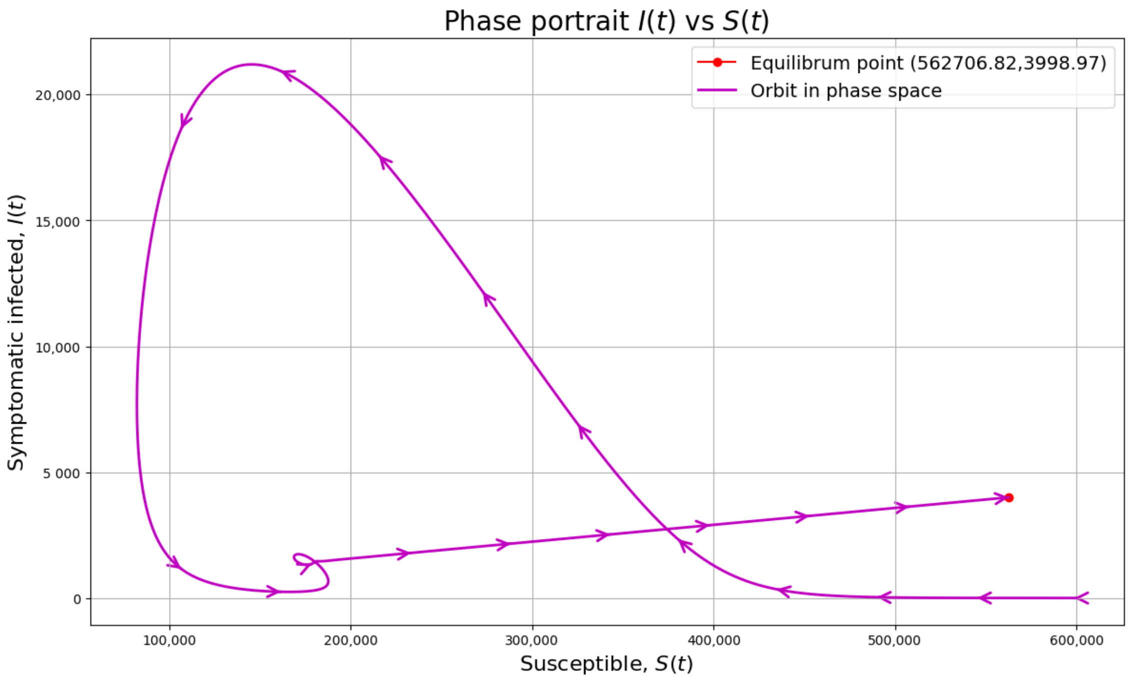

Figure S2. Phase portrait under endemic scenario, with R_0 = 1.2182.

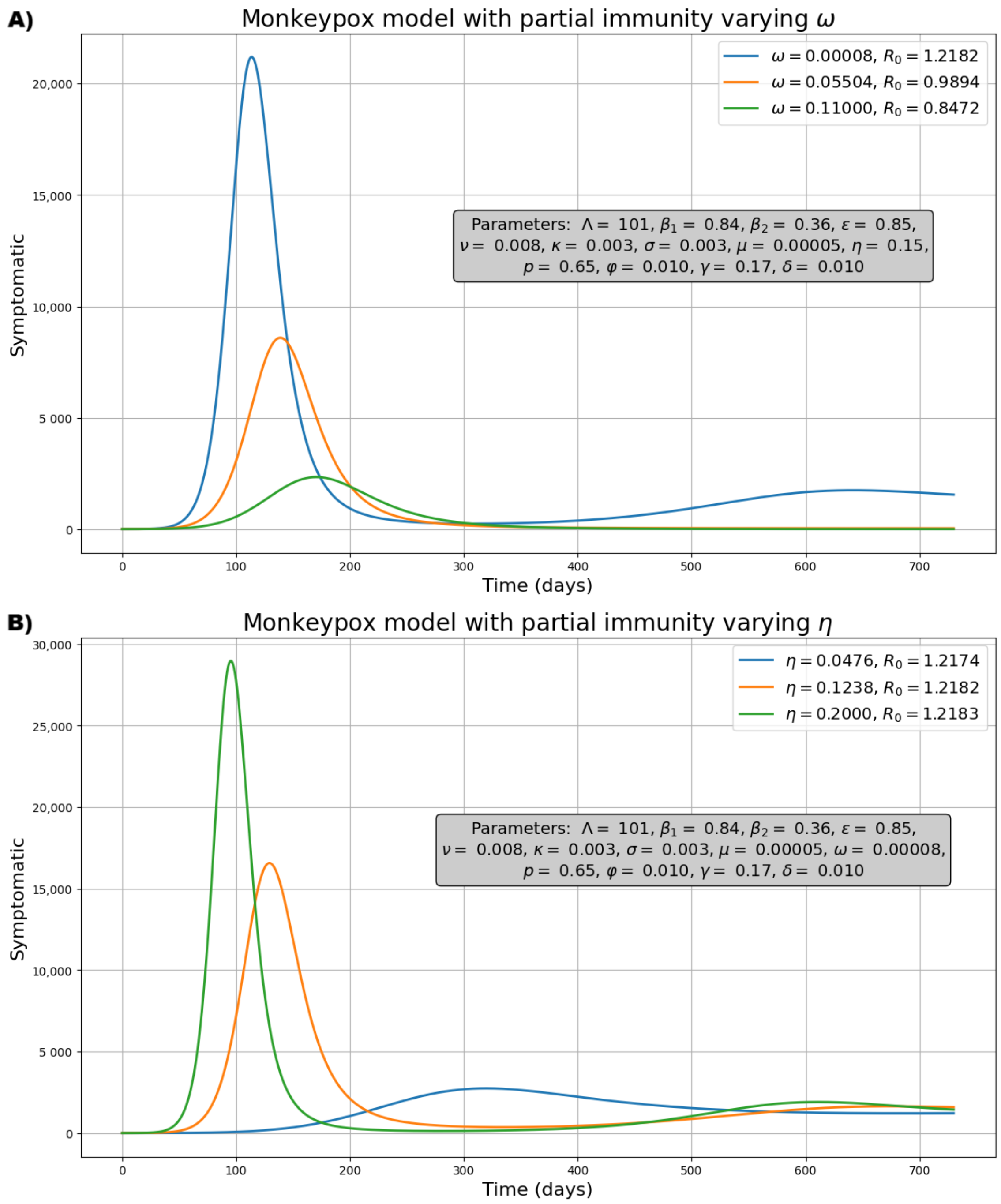

Figure S3. Effect of parameter variation on the dynamics of the symptomatic infected population: A) varying ω and, B) varying η.

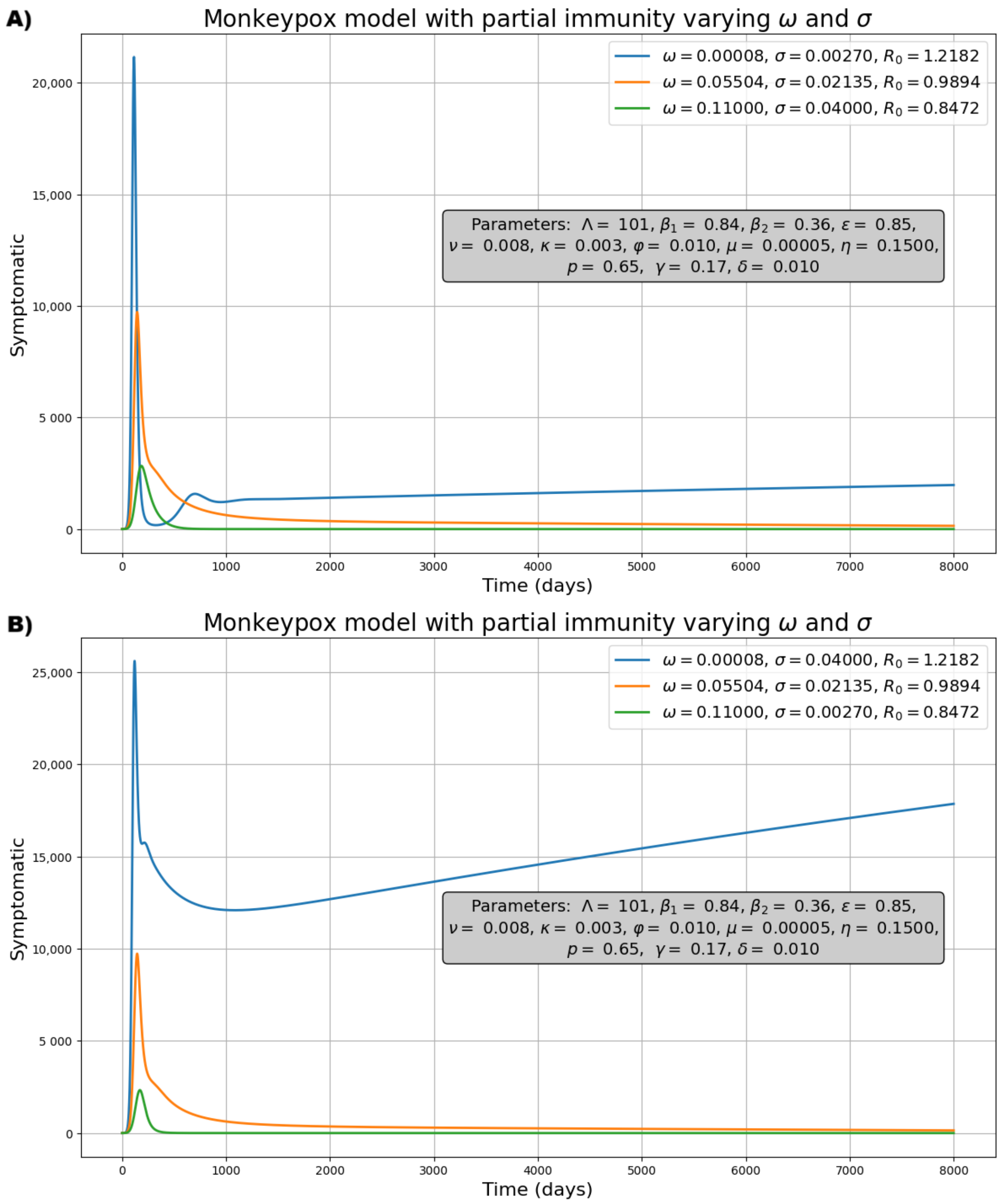

Figure S4. Dynamics of the symptomatic population: A) under the concurrent increase of ω and σ; and B) under the concurrent decrease of ω and σ.

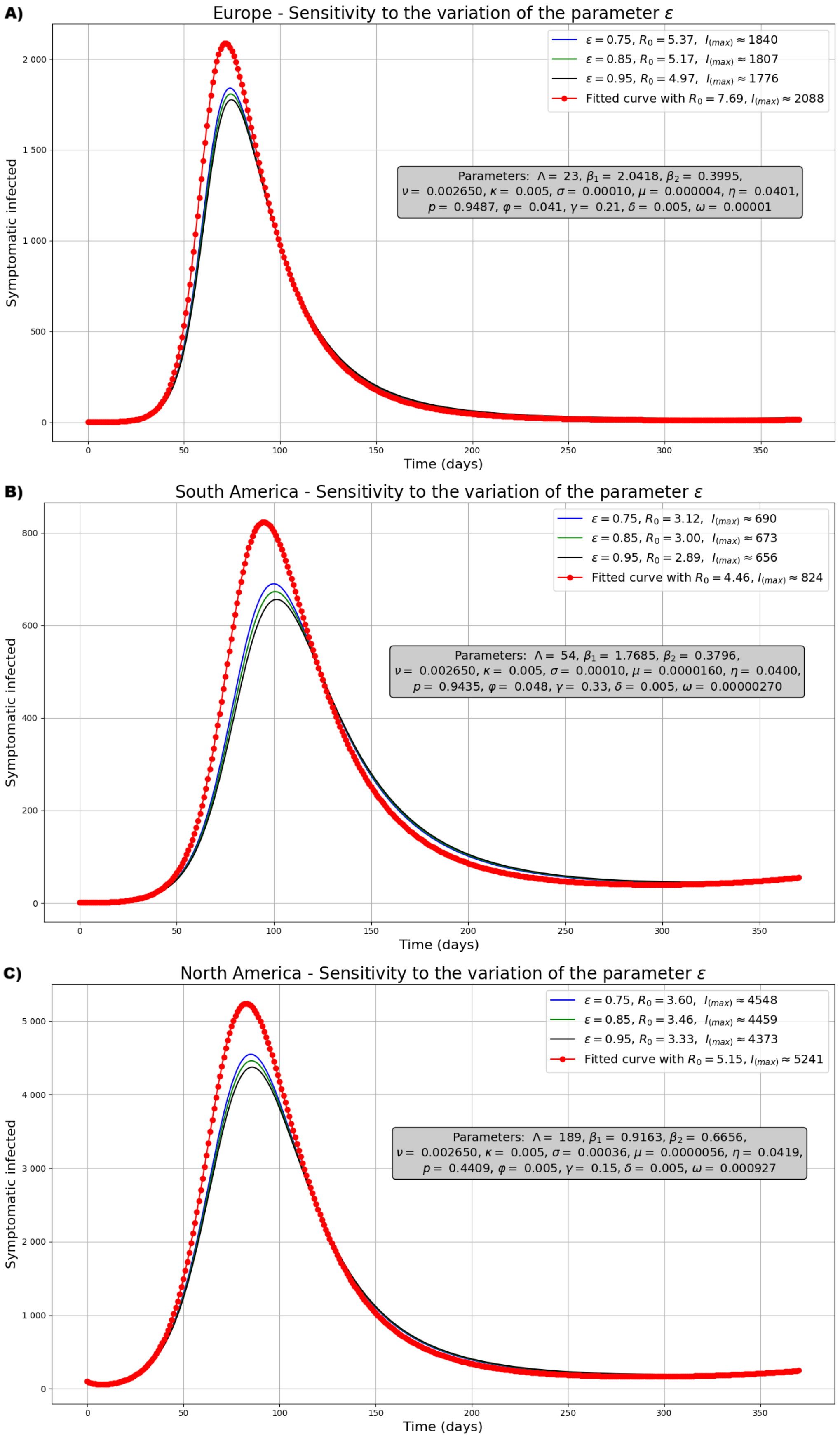

Figure S5. Effect of vaccination effectiveness (ε) variation on the infected population compared with the fitted curve without vaccination (with ε = 0, ν = 0, κ = 0) shown A) Europe, B) South America, C) North America.

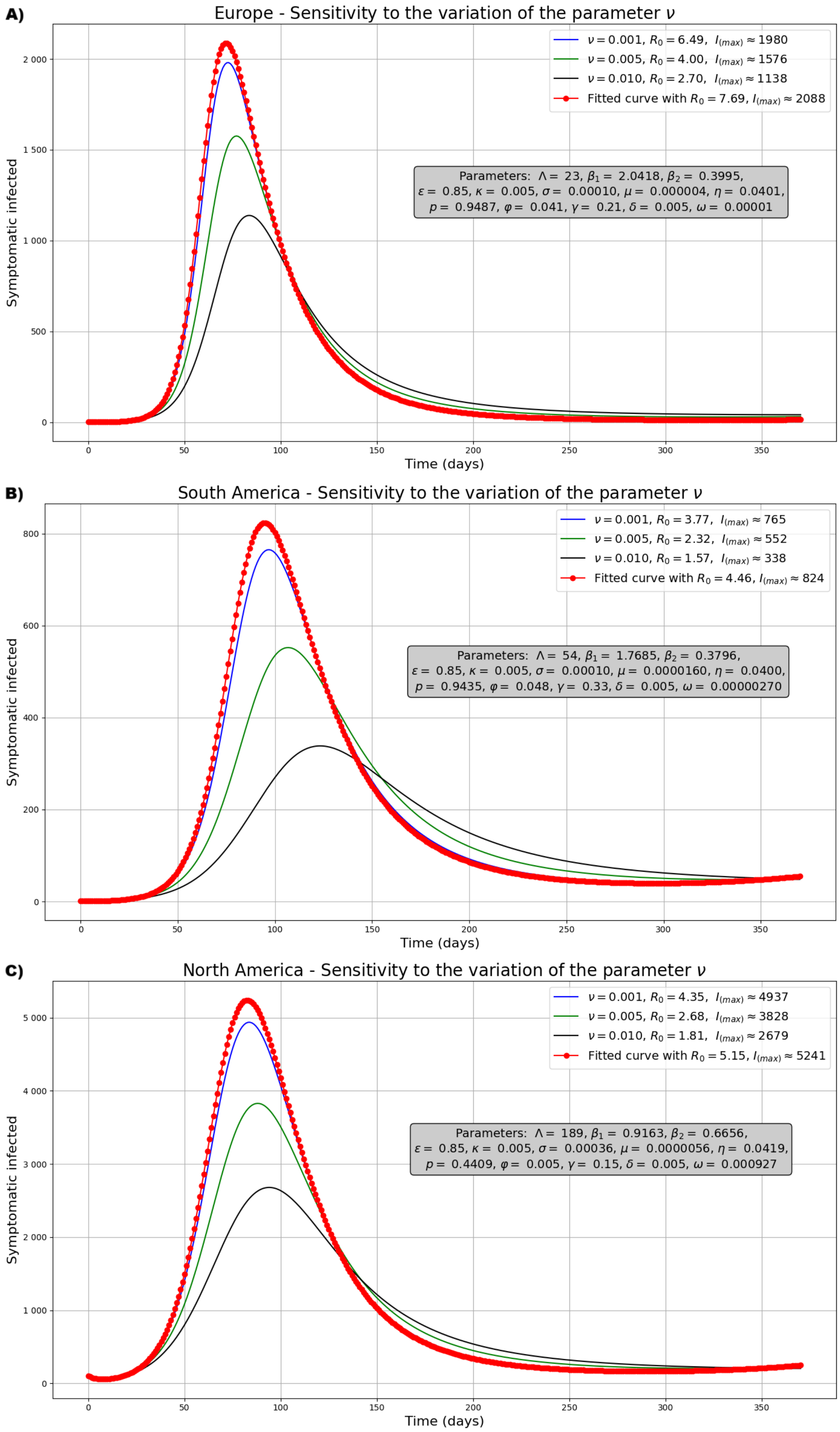

Figure S6. Effect of vaccination rate (ν) variation on the infected population compared with the fitted curve without vaccination (with ε = 0, ν = 0, κ = 0) shown for A) Europe, B) South America, C) North America.

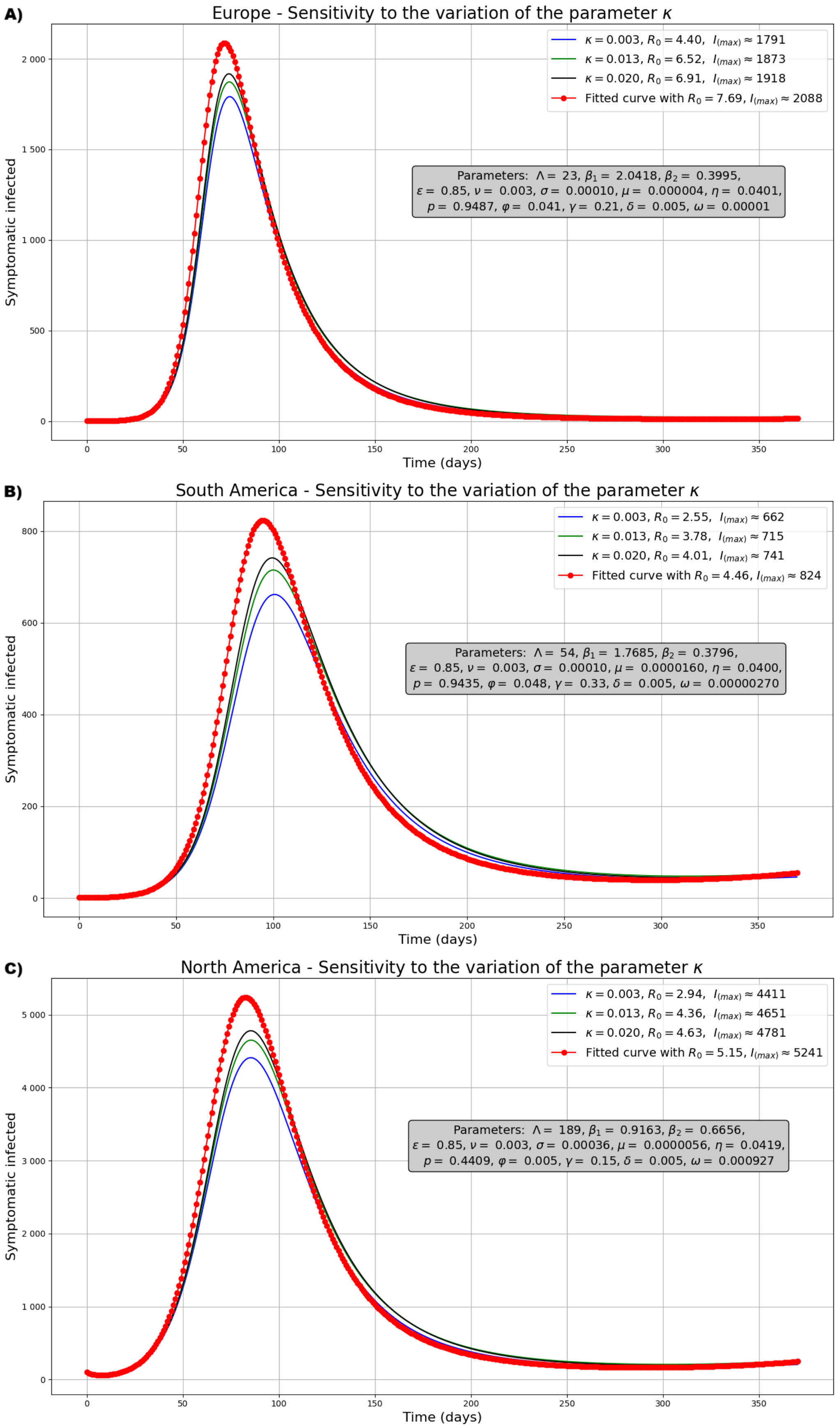

Figure S7. Effect of immunity lose rate (κ) variation on the infected population compared with the fitted curve without vaccination (with $\varepsilon = 0$, $\nu = 0$, $\kappa = 0$) shown for A) Europe, B) South America, C) North America.

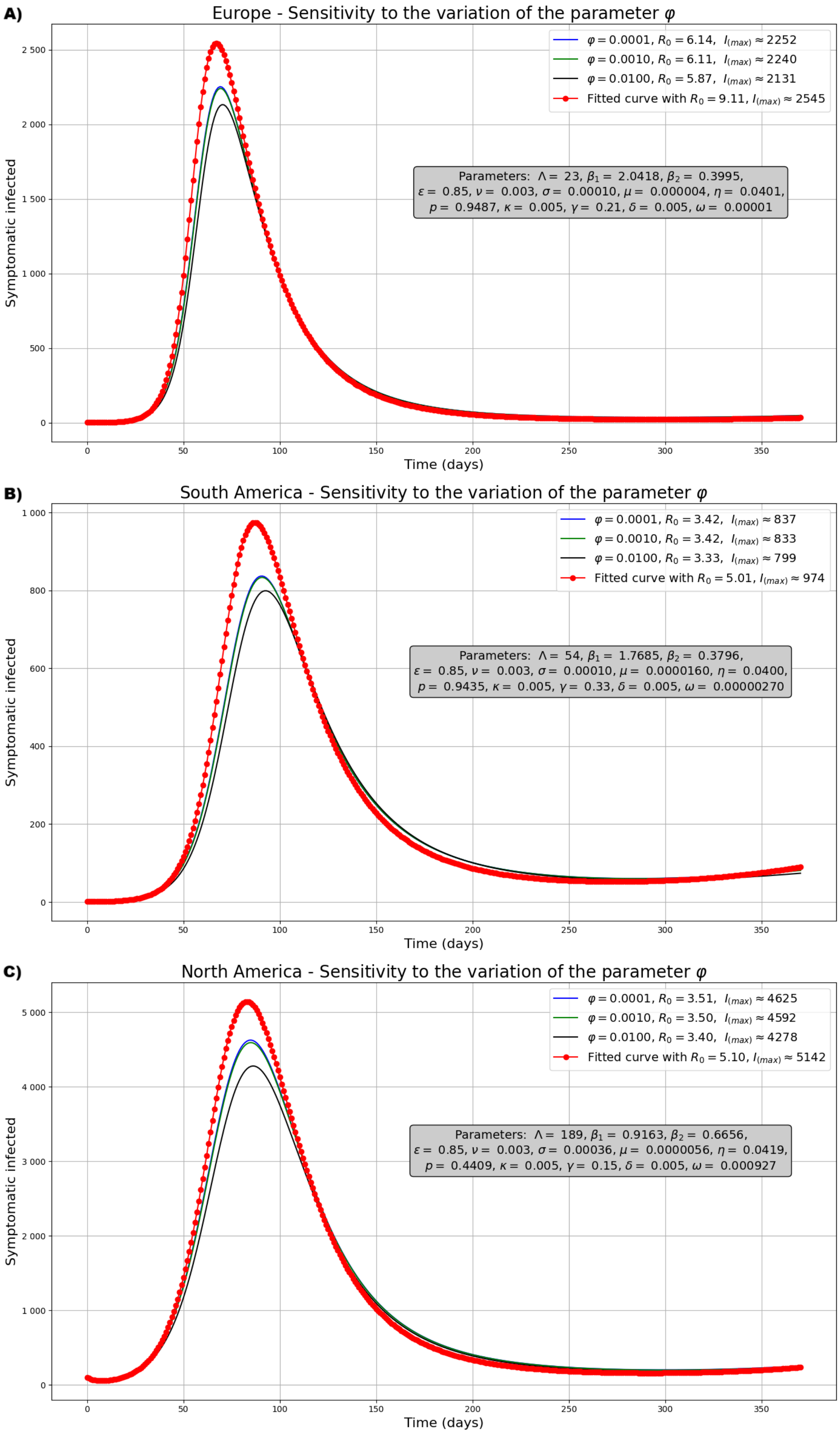

Figure S8. Effect of isolation rate (φ) variation and vaccination son the infected population compared with the fitted curve without vaccination (with $\varepsilon = 0$, $\nu = 0$, $\kappa = 0$) shown for A) Europe, B) South America, C) North America.
