# Supplementary material for: A Compartmental Mathematical Model to Assess the Impact of Vaccination, Isolation, and Key Epidemiological Parameters on Mpox Control
Source: Med Sci (Basel). 2025 Oct 10;13(4):226. doi: 10.3390/medsci13040226 (PMC12551027; doi:10.3390/medsci13040226)
Supplement: Supplementary file 1 [file medsci-13-00226-s001.zip › Supplementary material 2.pdf]

Supplementary material 2

Sensitivity analysis for $\mathcal{R}_0$

Table S1. Probability distributions of the parameters

Parameter	Probability distribution	Mean	Standard deviation
$\boldsymbol{\beta_1}$	Normal	$0.7\ day^{-1}$	$0.15\ day^{-1}$
$\boldsymbol{\beta_2}$	Normal	$0.45\ day^{-1}$	$0.15\ day^{-1}$
$\boldsymbol{\eta}$	Normal	$0.125\ day^{-1}$	$0.05\ day^{-1}$
$\boldsymbol{\varphi}$	Normal	$0.00679\ day^{-1}$	$0.00131\ day^{-1}$
$\boldsymbol{\gamma}$	Normal	$0.1176\ day^{-1}$	$0.03\ day^{-1}$
$\boldsymbol{\nu}$	Normal	$0.02\ day^{-1}$	$0.005\ day^{-1}$
$\boldsymbol{\varepsilon}$	Normal	0.85	$0.05\ day^{-1}$
$\boldsymbol{\kappa}$	Normal	$0.0045\ day^{-1}$	$0.0005\ day^{-1}$
$\boldsymbol{\mu}$	Normal	$3.013 \times 10^{-5}\ day^{-1}$	$0.5 \times 10^{-6}\ day^{-1}$
**Parameter**	**Probability distribution**	**Lower bound**	**Upper bound**
$\boldsymbol{p}$	Uniform	0.4	0.6
$\boldsymbol{\omega}$	Uniform	$0.00008\ day^{-1}$	$0.10992\ day^{-1}$

Table S2. Confidence intervals (CI) for the PRCC values of each parameter

Parameter	PRCC mean	Lower CI	Upper CI
$\boldsymbol{\beta_1}$	0.3181	0.2548	0.3791
$\boldsymbol{\beta_2}$	0.2671	0.2056	0.3234
$\boldsymbol{\eta}$	0.0153	-0.0406	0.0735
$\boldsymbol{\varphi}$	-0.0079	-0.0708	0.0502
$\boldsymbol{\gamma}$	-0.5102	-0.5504	-0.4668
$\boldsymbol{\nu}$	-0.4643	-0.5178	-0.4082
$\boldsymbol{\varepsilon}$	-0.1048	-0.1657	-0.0445
$\boldsymbol{\kappa}$	0.1883	0.1301	0.2471
$\boldsymbol{\mu}$	-0.0023	-0.0659	0.0574
$\boldsymbol{p}$	-0.0023	-0.0649	0.0603
$\boldsymbol{\omega}$	-0.3107	-0.3673	-0.2507

Global sensitivity analysis for the model

In this section, the estimated values of the indices are shown; the tables 3, 4 and 5 show the mean values of the indices obtained with a confidence level of 95%. In the case of S1 negative values close to zero are shown because the estimator used in the SALib package does not guarantee the positivity of these indices. In the case of S2 the values used for the creation of the heat map shown in the main text are shown; in the case of zero values, they were rounded from the negative indices (for example, the values of S2 for $(\boldsymbol{\sigma}, \boldsymbol{\mu})$ and $(\boldsymbol{\kappa}, \boldsymbol{\mu})$ without rounding are -0.00018 and -0.001927, respectively) since the confidence intervals are close to zero (that is, they are statistically indistinguishable from zero) and taking into account what was said for S1.

Table S3. First order Sobol sensitivity index (S1=S_j)

Parameter	S1	S1 confidence interval
$\boldsymbol{\Lambda}$	0.019461	0.002978
$\boldsymbol{\beta_1}$	0.008642	0.002566
$\boldsymbol{\beta_2}$	0.005051	0.00181
$\boldsymbol{\eta}$	0.046923	0.005179
$\boldsymbol{p}$	0.01647	0.003176
$\boldsymbol{\varphi}$	0.022593	0.004061
$\boldsymbol{\gamma}$	-1.3E-05	8.07E-05
$\boldsymbol{\delta}$	0.012119	0.004871
$\boldsymbol{\nu}$	0.026035	0.003357
$\boldsymbol{\varepsilon}$	0.00093	0.00125
$\boldsymbol{\sigma}$	0.017727	0.004296
$\boldsymbol{\kappa}$	0.005077	0.002016
$\boldsymbol{\mu}$	-3.6E-06	3.45E-06
$\boldsymbol{\omega}$	0.096067	0.01569
$\sum_{j=1}^{14} S_j =$	0.277078	

Table S4. Second order Sobol sensitivity index (S2=S_{ij})

Parameter interaction	S2	S2 confidence interval
$(\boldsymbol{\Lambda}, \boldsymbol{\beta_1})$	0.006163441	0.006020456
$(\boldsymbol{\Lambda}, \boldsymbol{\beta_2})$	0.004906686	0.006835301
$(\boldsymbol{\Lambda}, \boldsymbol{\eta})$	0.014831059	0.00677774
$(\boldsymbol{\Lambda}, \boldsymbol{p})$	0.007010326	0.00664115
$(\boldsymbol{\Lambda}, \boldsymbol{\varphi})$	0.009867964	0.006879949
$(\boldsymbol{\Lambda}, \boldsymbol{\gamma})$	0.003031413	0.006585451
$(\boldsymbol{\Lambda}, \boldsymbol{\delta})$	0.004535969	0.00809151
$(\boldsymbol{\Lambda}, \boldsymbol{\nu})$	0.011720371	0.007184071
$(\boldsymbol{\Lambda}, \boldsymbol{\varepsilon})$	0.003288296	0.006726357
$(\boldsymbol{\Lambda}, \boldsymbol{\sigma})$	0.013625962	0.010039782
$(\boldsymbol{\Lambda}, \boldsymbol{\kappa})$	0.003943973	0.007022687
$(\boldsymbol{\Lambda}, \boldsymbol{\mu})$	0.003066785	0.006602832
$(\boldsymbol{\Lambda}, \boldsymbol{\omega})$	0.024093693	0.008743734
$(\boldsymbol{\beta_1}, \boldsymbol{\beta_2})$	0.00263252	0.003643417
$(\boldsymbol{\beta_1}, \boldsymbol{\eta})$	0.004420641	0.003438676
$(\boldsymbol{\beta_1}, \boldsymbol{p})$	0.002589835	0.003582883
$(\boldsymbol{\beta_1}, \boldsymbol{\varphi})$	0.003442612	0.003783349
$(\boldsymbol{\beta_1}, \boldsymbol{\gamma})$	0.002335734	0.00372348
$(\boldsymbol{\beta_1}, \boldsymbol{\delta})$	0.004606059	0.004907721
$(\boldsymbol{\beta_1}, \boldsymbol{\nu})$	0.005299024	0.003842249
$(\boldsymbol{\beta_1}, \boldsymbol{\varepsilon})$	0.00283981	0.003796303
$(\boldsymbol{\beta_1}, \boldsymbol{\sigma})$	0.005358707	0.003844747
$(\boldsymbol{\beta_1}, \boldsymbol{\kappa})$	0.002238316	0.003626858
$(\boldsymbol{\beta_1}, \boldsymbol{\mu})$	0.002335492	0.003728061
$(\boldsymbol{\beta_1}, \boldsymbol{\omega})$	0.013165178	0.006151794
$(\boldsymbol{\beta_2}, \boldsymbol{\eta})$	0.00048116	0.002563786
$(\boldsymbol{\beta_2}, \boldsymbol{p})$	0.000547887	0.003081582

$(\boldsymbol{\beta}_2, \boldsymbol{\varphi})$	0.002158205	0.003298208
$(\boldsymbol{\beta}_2, \boldsymbol{\gamma})$	0	0.00271763
$(\boldsymbol{\beta}_2, \boldsymbol{\delta})$	0	0.003497158
$(\boldsymbol{\beta}_2, \boldsymbol{\nu})$	0.001694231	0.003141781
$(\boldsymbol{\beta}_2, \boldsymbol{\varepsilon})$	0.001199533	0.002711131
$(\boldsymbol{\beta}_2, \boldsymbol{\sigma})$	0.000970024	0.002842443
$(\boldsymbol{\beta}_2, \boldsymbol{\kappa})$	0.000964199	0.003011919
$(\boldsymbol{\beta}_2, \boldsymbol{\mu})$	0	0.002719828
$(\boldsymbol{\beta}_2, \boldsymbol{\omega})$	0	0.004561244
$(\boldsymbol{\eta}, \boldsymbol{p})$	0	0.007806288
$(\boldsymbol{\eta}, \boldsymbol{\varphi})$	0.004834101	0.007547502
$(\boldsymbol{\eta}, \boldsymbol{\gamma})$	0	0.00730147
$(\boldsymbol{\eta}, \boldsymbol{\delta})$	0.003166275	0.008877992
$(\boldsymbol{\eta}, \boldsymbol{\nu})$	0.008972084	0.008380777
$(\boldsymbol{\eta}, \boldsymbol{\varepsilon})$	0	0.007493832
$(\boldsymbol{\eta}, \boldsymbol{\sigma})$	0.002062744	0.007771053
$(\boldsymbol{\eta}, \boldsymbol{\kappa})$	0	0.007409877
$(\boldsymbol{\eta}, \boldsymbol{\mu})$	0	0.007304443
$(\boldsymbol{\eta}, \boldsymbol{\omega})$	0.019064759	0.016540256
$(\boldsymbol{p}, \boldsymbol{\varphi})$	0.002699544	0.005345002
$(\boldsymbol{p}, \boldsymbol{\gamma})$	0	0.004747948
$(\boldsymbol{p}, \boldsymbol{\delta})$	0	0.006520429
$(\boldsymbol{p}, \boldsymbol{\nu})$	0.004882636	0.00485955
$(\boldsymbol{p}, \boldsymbol{\varepsilon})$	0	0.004733408
$(\boldsymbol{p}, \boldsymbol{\sigma})$	0.002362038	0.00553096
$(\boldsymbol{p}, \boldsymbol{\kappa})$	0	0.004897347
$(\boldsymbol{p}, \boldsymbol{\mu})$	0	0.004751529
$(\boldsymbol{p}, \boldsymbol{\omega})$	0.011680671	0.007838432
$(\boldsymbol{\varphi}, \boldsymbol{\gamma})$	0.0038937	0.006502873
$(\boldsymbol{\varphi}, \boldsymbol{\delta})$	0.01305595	0.008329664
$(\boldsymbol{\varphi}, \boldsymbol{\nu})$	0.012740211	0.007037575
$(\boldsymbol{\varphi}, \boldsymbol{\varepsilon})$	0.003832377	0.006375674
$(\boldsymbol{\varphi}, \boldsymbol{\sigma})$	0.009274996	0.008473141
$(\boldsymbol{\varphi}, \boldsymbol{\kappa})$	0.003883641	0.006928645
$(\boldsymbol{\varphi}, \boldsymbol{\mu})$	0.003896744	0.006507624
$(\boldsymbol{\varphi}, \boldsymbol{\omega})$	0.025435394	0.008221849
$(\boldsymbol{\gamma}, \boldsymbol{\delta})$	0	0.00023639
$(\boldsymbol{\gamma}, \boldsymbol{\nu})$	4.65677E-05	0.000162778
$(\boldsymbol{\gamma}, \boldsymbol{\varepsilon})$	3.28994E-05	0.00015044
$(\boldsymbol{\gamma}, \boldsymbol{\sigma})$	8.29216E-05	0.000173055
$(\boldsymbol{\gamma}, \boldsymbol{\kappa})$	4.75866E-05	0.000146109
$(\boldsymbol{\gamma}, \boldsymbol{\mu})$	4.61294E-05	0.000155459
$(\boldsymbol{\gamma}, \boldsymbol{\omega})$	0	0.000209603
$(\boldsymbol{\delta}, \boldsymbol{\nu})$	0	0.007231837
$(\boldsymbol{\delta}, \boldsymbol{\varepsilon})$	0	0.007276809
$(\boldsymbol{\delta}, \boldsymbol{\sigma})$	0	0.008360351
$(\boldsymbol{\delta}, \boldsymbol{\kappa})$	0	0.007448487
$(\boldsymbol{\delta}, \boldsymbol{\mu})$	0	0.007380276
$(\boldsymbol{\delta}, \boldsymbol{\omega})$	0.005691744	0.008967763
$(\boldsymbol{\nu}, \boldsymbol{\varepsilon})$	0.000673784	0.004501999
$(\boldsymbol{\nu}, \boldsymbol{\sigma})$	0.006956533	0.005006446
$(\boldsymbol{\nu}, \boldsymbol{\kappa})$	0.002201136	0.004551978

(ν, μ)	0.00061692	0.004502173
(ν, ω)	0.02724038	0.007174984
(ε, σ)	0.000471753	0.002039379
(ε, κ)	0.000181521	0.001708843
(ε, μ)	0.000437393	0.001692511
(ε, ω)	0.001733868	0.002065843
(σ, κ)	1.05179E-05	0.006855339
(σ, μ)	0	0.006587315
(σ, ω)	0.054326513	0.016354699
(κ, μ)	0	0.002683445
(κ, ω)	0.005886654	0.004670958
(μ, ω)	1.72304E-05	3.44144E-05

Table S5. Total Sobol sensitivity index (ST=ST_j)

Parameter	ST	ST confidence interval
Λ	0.201708	0.011025
β_1	0.146427	0.012752
β_2	0.059806	0.004892
η	0.34157	0.024395
p	0.174164	0.013819
φ	0.291414	0.019247
γ	0.000183	7.98E-05
δ	0.206852	0.019209
ν	0.289137	0.023184
ε	0.034473	0.002638
σ	0.270371	0.02042
κ	0.077038	0.008156
μ	6.29E-07	1.88E-07
ω	0.577836	0.03103
$\sum_{j=1}^{14} ST_j =$	2.670981	

Case study: Parameter estimation and model fitting with rounded values for initial conditions

Table S6. Range parameter's values and initial conditions for the model fitting - World

Parameter	Initial guess	Range [min,max] (Unit)	Fitted value
$\boldsymbol{S(0)}$	160000	$[1.0\times10^4, 2.0\times10^6]\ persons$	94854.1383
$\boldsymbol{I(0)}$	27	$[27,28]\ persons$	27
$\boldsymbol{\Lambda}$	34	$[1,3231] person \times day^{-1}$	7.73970439
$\boldsymbol{\beta_1}$	1.2	$[0.4,2.5]\ day^{-1}$	1.67953368
$\boldsymbol{\beta_2}$	0.8	$[0.1,2.5]\ day^{-1}$	0.17020184
$\boldsymbol{\eta}$	0.08	$[0.01, 0.2]\ day^{-1}$	0.02295726
$\boldsymbol{p}$	0.46	$[0,1]$	0.96584613
$\boldsymbol{\varphi}$	0.006	$[0.00001,0.01]\ day^{-1}$	0.00717407
$\boldsymbol{\gamma}$	0.06	$[0.0357,0.3333]\ day^{-1}$	0.25943508
$\boldsymbol{\delta}$	0.005	$[0.001,0.05]\ day^{-1}$	0.00494816
$\boldsymbol{\sigma}$	0.003	$[0.0001,0.04]\ day^{-1}$	1.6023×10^{-4}
$\boldsymbol{\mu}$	0.00002	$[1.0\times10^{-6}, 1.0\times10^{-4}]\ day^{-1}$	2.7312×10^{-5}
$\boldsymbol{\omega}$	0.00004	$[0.00001, 0.11]\ day^{-1}$	0.00237251
$\boldsymbol{\rho}$	0.7	$[0.1,0.95]$	0.93219423

Table S7. Range parameter's values and initial conditions for the model fitting - Europe

Parameter	Initial guess	Range [min,max] (Unit)	Fitted value
$\boldsymbol{S(0)}$	25000	$[1.0,2.0\times10^5]\ persons$	26132.5978
$\boldsymbol{I(0)}$	1	$[1,1.1]\ persons$	1
$\boldsymbol{\Lambda}$	30	$[1,3231] person \times day^{-1}$	22.6202113
$\boldsymbol{\beta_1}$	1.2	$[0.4,2.5]\ day^{-1}$	2.04177890
$\boldsymbol{\beta_2}$	0.8	$[0.1,2.5]\ day^{-1}$	0.39950298
$\boldsymbol{\eta}$	0.08	$[0.04, 0.2]\ day^{-1}$	0.04005441
$\boldsymbol{p}$	0.46	$[0.1,1]$	0.94874630
$\boldsymbol{\varphi}$	0.03	$[0.00001,0.09]\ day^{-1}$	0.04094240
$\boldsymbol{\gamma}$	0.06	$[0.0357,0.3333]\ day^{-1}$	0.21399404
$\boldsymbol{\delta}$	0.005	$[0.0001,0.01]\ day^{-1}$	0.00469751
$\boldsymbol{\sigma}$	0.003	$[0.0001,0.04]\ day^{-1}$	1.0153×10^{-4}
$\boldsymbol{\mu}$	0.000005	$[2.73973\times10^{-6}, 9.0\times10^{-4}]\ day^{-1}$	4.3514×10^{-6}
$\boldsymbol{\omega}$	0.00004	$[1\times10^{-7}, 0.3]\ day^{-1}$	1.4828×10^{-5}
$\boldsymbol{\rho}$	0.6	$[0.1,0.95]$	0.91519545

Table S8. Range parameter's values and initial conditions for the model fitting – South America

Parameter	Initial guess	Range [min,max] (Unit)	Fitted value
$\boldsymbol{S(0)}$	25000	$[1.0,2.0\times10^5]\ persons$	16330.4531
$\boldsymbol{I(0)}$	2	$[2,2.1]\ persons$	2
$\boldsymbol{\Lambda}$	30	$[1,3231] person \times day^{-1}$	54.2253827
$\boldsymbol{\beta_1}$	1.2	$[0.4,2.5]\ day^{-1}$	1.76850088
$\boldsymbol{\beta_2}$	0.8	$[0.1,2.5]\ day^{-1}$	0.37955379
$\boldsymbol{\eta}$	0.08	$[0.04, 0.2]\ day^{-1}$	0.04000883
$\boldsymbol{p}$	0.46	$[0.1,1]$	0.94346047
$\boldsymbol{\varphi}$	0.03	$[0.00001,0.09]\ day^{-1}$	0.04788072
$\boldsymbol{\gamma}$	0.06	$[0.0357,0.3333]\ day^{-1}$	0.33142887
$\boldsymbol{\delta}$	0.005	$[0.00001,0.01]\ day^{-1}$	0.00455631

$\boldsymbol{\sigma}$	0.003	$[0.0001,0.04]\ day^{-1}$	1.0003×10^{-4}
$\boldsymbol{\mu}$	0.000005	$[2.73973 \times 10^{-6}, 9.0 \times 10^{-4}]\ day^{-1}$	1.6049×10^{-5}
$\boldsymbol{\omega}$	0.00004	$[1 \times 10^{-7}, 0.3]\ day^{-1}$	2.7038×10^{-6}
$\boldsymbol{\rho}$	0.6	$[0.1, 0.95]$	0.94531585

Table S9. Range parameter's values and initial conditions for the model fitting – North America

Parameter	Initial guess	Range [min,max] (Unit)	Fitted value
$\boldsymbol{S}(\mathbf{0})$	25000	$[1.0,5.0 \times 10^{4}]\ persons$	103602.999
$\boldsymbol{I}(\mathbf{0})$	103	$[103,103.1]\ persons$	103
$\boldsymbol{\Lambda}$	15	$[1,3231] person \times day^{-1}$	189.385924
$\boldsymbol{\beta_1}$	0.9	$[0.4,1.0]\ day^{-1}$	0.91634522
$\boldsymbol{\beta_2}$	0.8	$[0.1,1.0]\ day^{-1}$	0.66563355
$\boldsymbol{\eta}$	0.05	$[0.04, 0.2]\ day^{-1}$	0.04191390
$\boldsymbol{p}$	0.46	$[0.1,1]$	0.44090917
$\boldsymbol{\varphi}$	0.006	$[0.00001,0.01]\ day^{-1}$	0.00466125
$\boldsymbol{\gamma}$	0.06	$[0.0357,0.3333]\ day^{-1}$	0.14785275
$\boldsymbol{\delta}$	0.005	$[0.00001,0.01]\ day^{-1}$	0.00494974
$\boldsymbol{\sigma}$	0.003	$[0.0001,0.04]\ day^{-1}$	3.6366×10^{-4}
$\boldsymbol{\mu}$	0.000005	$[2.73973 \times 10^{-6}, 6.0274 \times 10^{-5}]\ day^{-1}$	5.5609×10^{-6}
$\boldsymbol{\omega}$	0.00004	$[1 \times 10^{-7}, 0.11]\ day^{-1}$	9.2659×10^{-4}
$\boldsymbol{\rho}$	0.6	$[0.1, 0.95]$	0.61892306
