# Supplementary material for: A Compartmental Mathematical Model to Assess the Impact of Vaccination, Isolation, and Key Epidemiological Parameters on Mpox Control
Source: Med Sci (Basel). 2025 Oct 10;13(4):226. doi: 10.3390/medsci13040226 (PMC12551027; doi:10.3390/medsci13040226)
Supplement: Supplementary file 1 [file medsci-13-00226-s001.zip › Supplementary Material 3.pdf]

Local stability for the endemic equilibrium point

The Jacobian matrix for this point can be written as follow:

$$J(P^*) = \begin{pmatrix} -(\beta_1 i + \beta_2 a) - \varepsilon v - \mu & 0 & -\beta_1 s & -\beta_2 s & 0 & \sigma & \kappa \\ \beta_1 i + \beta_2 a & -(\eta + \mu) & \beta_1 s & \beta_2 s & 0 & 0 & 0 \\ 0 & p\eta & -(\varphi + \gamma + \omega + \mu) & 0 & 0 & 0 & 0 \\ 0 & (1-p)\eta & 0 & -(\gamma + \mu) & 0 & 0 & 0 \\ 0 & 0 & \varphi & 0 & -(\delta + \omega + \mu) & 0 & 0 \\ 0 & 0 & \gamma & \gamma & \delta & -(\sigma + \mu) & 0 \\ \varepsilon v & 0 & 0 & 0 & 0 & 0 & -(\kappa + \mu) \end{pmatrix}$$

Where

$$i = \frac{I^*}{N^*}, a = \frac{A^*}{N^*}, s = \frac{S^*}{N^*}$$

And the characteristic equation is

$$b_0\lambda^7 + b_1\lambda^6 + b_2\lambda^5 + b_3\lambda^4 + b_4\lambda^3 + b_5\lambda^2 + b_6\lambda + b_7 = 0$$

Which coefficients are

$$b_0 = 1$$

$$b_1 = a\beta_2 + \beta_1 i + \varepsilon v + \delta + \eta + 2\gamma + \kappa + 7\mu + 2\omega + \sigma + \varphi$$

$$\begin{aligned} b_2 = {} & (2\gamma + 2\omega + 6\mu + (a + (p-1)s)\beta_2 + \varphi + \delta + \sigma + (-ps + i)\beta_1 + \varepsilon v + \kappa)\eta + \gamma^2 \\ & + (2a\beta_2 + 2\beta_1 i + 2\varepsilon v + 2\delta + 2\kappa + 12\mu + 3\omega + 2\sigma + \varphi)\gamma + \omega^2 \\ & + (2a\beta_2 + 2\beta_1 i + 2\varepsilon v + \delta + 2\kappa + 12\mu + 2\sigma + \varphi)\omega + 21\mu^2 \\ & + (6a\beta_2 + 6\beta_1 i + 6\varepsilon v + 6\delta + 6\kappa + 6\sigma + 6\varphi)\mu + a(\kappa + \sigma + \varphi + \delta)\beta_2 \\ & + (\beta_1 i + \varepsilon v + \delta + \kappa + \sigma)\varphi + (\beta_1 i + \varepsilon v + \kappa + \sigma)\delta + (\beta_1 i + \varepsilon v + \kappa)\sigma \\ & + i\kappa\beta_1 \end{aligned}$$

$$\begin{aligned} b_3 = {} & (15\mu^2 + (10\gamma + 10\omega + (5a + (5p-5)s)\beta_2 + 5\sigma + 5\delta + 5\varphi + 5\kappa + (-5ps + 5i)\beta_1 \\ & + 5\varepsilon v)\mu + \gamma^2 \\ & + (3\omega + (2a + (p-1)s)\beta_2 + 2\sigma + 2\delta + \varphi + 2\kappa + (-ps + 2i)\beta_1 + 2\varepsilon v)\gamma \\ & + \omega^2 + ((2a + (2p-2)s)\beta_2 + 2\sigma + \delta + \varphi + 2\kappa + (-ps + 2i)\beta_1 + 2\varepsilon v)\omega \\ & + ((a + (p-1)s)\sigma + (a + (p-1)s)\delta + (a + (p-1)s)\varphi \\ & + (a + (p-1)s)\kappa + \varepsilon sv(p-1))\beta_2 + (\delta + \varphi + (-ps + i)\beta_1 + \varepsilon v + \kappa)\sigma \\ & + (\varphi + (-ps + i)\beta_1 + \varepsilon v + \kappa)\delta + (\beta_1 i + \varepsilon v + \kappa)\varphi + \beta_1((-ps + i)\kappa \\ & - \varepsilon psv))\eta + 35\mu^3 + (15\beta_2 a + 15\beta_1 i + 15\varepsilon v + 15\delta + 30\gamma + 15\kappa + 30\omega \\ & + 15\sigma + 15\varphi)\mu^2 + (5\gamma^2 + (10\beta_2 a + 10\beta_1 i + 10\varepsilon v + 10\delta + 10\kappa + 15\omega \\ & + 10\sigma + 5\varphi)\gamma + 5\omega^2 + (10\beta_2 a + 10\beta_1 i + 10\varepsilon v + 5\delta + 10\kappa + 10\sigma + 5\varphi)\omega \\ & + 5a(\kappa + \sigma + \varphi + \delta)\beta_2 + (5\beta_1 i + 5\varepsilon v + 5\delta + 5\kappa + 5\varphi)\sigma \\ & + (5\beta_1 i + 5\varepsilon v + 5\kappa + 5\varphi)\delta + (5\beta_1 i + 5\varepsilon v + 5\kappa)\varphi + 5i\kappa\beta_1)\mu \\ & + (\beta_2 a + \beta_1 i + \varepsilon v + \delta + \kappa + \omega + \sigma)\gamma^2 \\ & + \Big(\omega^2 + (3\beta_2 a + 3\beta_1 i + 3\varepsilon v + \delta + 3\kappa + 3\sigma + \varphi)\omega + 2\Big(\kappa + \sigma + \frac{\varphi}{2} \\ & + \delta)a\beta_2 + (2\beta_1 i + 2\varepsilon v + 2\delta + 2\kappa + \varphi)\sigma + (2\beta_1 i + 2\varepsilon v + 2\kappa + \varphi)\delta \\ & + (\beta_1 i + \varepsilon v + \kappa)\varphi + 2i\kappa\beta_1)\gamma + (\beta_2 a + \beta_1 i + \varepsilon v + \kappa + \sigma)\omega^2 \\ & + \Big(2\Big(\kappa + \sigma + \frac{\varphi}{2} + \frac{\delta}{2}\Big)a\beta_2 + (2\beta_1 i + 2\varepsilon v + \delta + 2\kappa + \varphi)\sigma + (\beta_1 i + \varepsilon v + \kappa)\delta \\ & + (\beta_1 i + \varepsilon v + \kappa)\varphi + 2i\kappa\beta_1)\omega + a((\kappa + \varphi + \delta)\sigma + (\kappa + \varphi)\delta + \kappa\varphi)\beta_2 \\ & + \big((\beta_1 i + \varepsilon v + \kappa + \varphi)\delta + (\beta_1 i + \varepsilon v + \kappa)\varphi + i\kappa\beta_1)\sigma \\ & + \big((\beta_1 i + \varepsilon v + \kappa)\varphi + i\kappa\beta_1\big)\delta + i\kappa\varphi\beta_1 \end{aligned}$$

$$
\begin{aligned}
b_4 = {} & (20\mu^3 + (20\gamma + 20\omega + 10\sigma + ((10p - 10)s + 10a)\beta_2 + 10\delta + 10\kappa + 10\varphi + (-10ps \\
& + 10i)\beta_1 + 10\varepsilon v)\mu^2 \\
& + (4\gamma^2 + (12\omega + 8\sigma + ((4p - 4)s + 8a)\beta_2 + 8\delta + 8\kappa + 4\varphi + (-4ps \\
& + 8i)\beta_1 + 8\varepsilon v)\gamma + 4\omega^2 + (8\sigma \\
& + ((8p - 8)s + 8a)\beta_2 + 4\delta + 8\kappa + 4\varphi + (-4ps + 8i)\beta_1 + 8\varepsilon v)\omega \\
& + (((4p - 4)s + 4a)\beta_2 + 4\delta + 4\kappa + 4\varphi + (-4ps + 4i)\beta_1 + 4\varepsilon v)\sigma + (((4p \\
& - 4)s + 4a)\delta + ((4p - 4)s + 4a)\kappa + ((4p - 4)s + 4a)\varphi + 4\varepsilon sv(p\text{-}1)\,\beta_2 \\
& + (4\kappa + 4\varphi + (-4ps + 4i)\beta_1 + 4\varepsilon v)\delta + (4\varphi \\
& + (-4ps + 4i)\beta_1)\kappa + (4\beta_1 i + 4\varepsilon v)o - 4\varepsilon\beta_1 psv)\mu \\
& + (\beta_2 a + \beta_1 i + \varepsilon v + \delta + \kappa + \omega + \sigma)\gamma^2 \\
& + (\omega^2 + (3\sigma + ((p - 1)s + 3a)\beta_2 + \delta + 3\kappa + \varphi + (-ps + 3i)\beta_1 + 3\varepsilon v)\omega \\
& + \big((a + (p - 1)s)\beta_2 + 2\delta + 2\kappa + \varphi + (-ps + i)\beta_1 + 2\varepsilon v)\sigma + ((2a + (p \\
& - 1)s)\delta + (2a + (p - 1)s)\kappa + a\varphi + \varepsilon sv(p - 1))\beta_2 \\
& + (\varphi + 2\kappa + (-ps + 2i)\beta_1 + 2\varepsilon v)\delta \\
& + (\varphi + (-ps + 2i)\beta_1)\kappa + (\beta_1 i + \varepsilon v)\varphi - \varepsilon\beta_1 psv)\gamma \\
& + (\sigma + (a + (p - 1)s)\beta_2 + \varepsilon v + \beta_1 i + \kappa)\omega^2 \\
& + \Big(\big((2a + (2p - 2)s)\beta_2 + \delta + \varphi + 2\kappa + (-ps + 2i)\beta_1 + 2\varepsilon v\big)\sigma \\
& + \big((a + (p - 1)s)\delta + (2a + (2p - 2)s)\kappa + (a + (p - 1)s)\varphi \\
& + 2\varepsilon sv(p - 1)\big)\,\beta_2 + (\beta_1 i + \varepsilon v + \kappa)\delta + (\varphi + (-ps + 2i)\beta_1)\kappa \\
& + (\beta_1 i + \varepsilon v)\varphi - \varepsilon\beta_1 psv)\omega + (((a + (p - 1)s)\delta + (a + (p - 1)s)\varphi + (a \\
& + (p - 1)s)\kappa + \varepsilon sv(p - 1))\beta_2 + (\varphi + (-ps + i)\beta_1 + \varepsilon v + \kappa)\delta \\
& + (\varphi + (-ps + i)\beta_1)\kappa + (\beta_1 i + \varepsilon v)\varphi - \varepsilon\beta_1 psv)\sigma + (((a + (p - 1)s)\varphi + (a \\
& + (p - 1)s)\kappa + \varepsilon sv(p - 1))\delta + ((a + (p - 1)s)\kappa + \varepsilon sv(p - 1))\varphi)\beta_2 \\
& + \big((\varphi + (-ps + i)\beta_1)\kappa + (\beta_1 i + \varepsilon v)\phi - \varepsilon\beta_1 psv)\delta + i\kappa\varphi\beta_1)\eta + 35\mu^4 \\
& + (20\beta_2 a + 20\beta_1 i + 20\varepsilon v + 20\delta + 40\gamma + 20\kappa + 40\omega + 20\sigma + 20\varphi)\mu^3 \\
& + (10\gamma^2 + (20\beta_2 a + 20\beta_1 i + 20\varepsilon\text{v} + 20\delta + 20\kappa + 30\omega + 20\sigma + 10\varphi)\gamma \\
& + 10\omega^2 + (20\beta_2 a + 20\beta_1 i + 20\varepsilon\text{v} + 10\delta + 20\kappa + 20\sigma + 10\varphi)\omega \\
& + (10\beta_2 a + 10\beta_1 i + 10\varepsilon v + 10\delta + 10\kappa + 10\varphi)\sigma + 10a(\kappa + \varphi + \delta)\beta_2 \\
& + (10\beta_1 i + 10\varepsilon v + 10\kappa + 10\varphi)\delta + (10\beta_1 i + 10\varphi)\kappa + 10(\beta_1 i + \varepsilon v)\varphi)\mu^2 \\
& + \big((4\beta_2 a + 4\beta_1 i + 4\varepsilon v + 4\delta + 4\kappa + 4\omega + 4\sigma)\gamma^2 \\
& + (4\omega^2 + (12\beta_2 a + 12\beta_1 i + 12\varepsilon v + 4\delta + 12\kappa + 12\sigma + 4\varphi)\omega \\
& + (8\beta_2 a + 8\beta_1 i + 8\varepsilon v + 8\delta + 8\kappa + 4\varphi)\sigma + 8a(\kappa + \frac{\varphi}{2} + \delta\big)\,\beta_2 \\
& + (8\beta_1 i + 8\varepsilon v + 8\kappa + 4\varphi)\delta + (8\beta_1 i + 4\varphi)\kappa + 4(\beta_1 i + \varepsilon v)\varphi)\gamma \\
& + (4\beta_2 a + 4\beta_1 i + 4\varepsilon v + 4\kappa + 4\sigma)\omega^2 + \big((8\beta_2 a + 8\beta_1 i + 8\varepsilon v + 4\delta + 8\kappa \\
& + 4\varphi)\sigma + 8\left(\kappa + \frac{\varphi}{2} + \frac{\delta}{2}\right)a\beta_2 \\
& + (4\beta_1 i + 4\varepsilon v + 4\kappa)\delta + (8\beta_1 i + 4\varphi)\kappa + 4(\beta_1 i + \varepsilon v)\varphi)\omega \\
& + (4a(\kappa + \varphi + \delta)\beta_2 + (4\beta_1 i + 4\varepsilon v + 4\kappa + 4\varphi)\delta + (4\beta_1 i + 4\varphi)\kappa \\
& + 4(\beta_1 i + \varepsilon v)\varphi)\sigma + 4a((\kappa \\
& + \varphi)\delta + \kappa\varphi)\beta_2 + \big((4\beta_1 i + 4\varphi)\kappa + 4(\beta_1 i + \varepsilon v)\varphi\big)\delta + 4i\kappa\varphi\beta_1\big)\mu \\
& + \big((\beta_2 a + \beta_1 i + \varepsilon v + \kappa + \sigma)\omega + (\beta_2 a + \beta_1 i + \varepsilon\text{v} + \delta + \kappa)\sigma + a(\kappa + \delta)\beta_2 \\
& + (\beta_1 i + \varepsilon v + \kappa)\delta + i\kappa\beta_1)\gamma^2 \\
& + \Big((\beta_2 a + \beta_1 i + \varepsilon v + \kappa + \sigma)\omega^2 + \big((3\beta_2 a + 3\beta_1 i + 3\varepsilon v + \delta + 3\kappa + \varphi)\sigma \\
& + 3\left(\kappa + \frac{\varphi}{3} + \frac{\delta}{3}\right)a\beta_2 + (\beta_1 i + \varepsilon v + \kappa)\delta + (3\beta_1 i + \varphi)\kappa + (\beta_1 i + \varepsilon v)\varphi)\omega \\
& + \left(2a\left(\kappa + \frac{\varphi}{2} + \delta\right)\beta_2 + (2\beta_1 i + 2\varepsilon v + 2\kappa + \varphi)\delta + (2\beta_1 i + \varphi)\kappa\right.
\end{aligned}
$$

$$+ (\beta_1 i + \varepsilon v)\varphi)\, \sigma + ((2\kappa + \varphi)\delta$$
$$+ \kappa\varphi)a\beta_2 + \big((2\beta_1 i + \varphi)\kappa + (\beta_1 i + \varepsilon v)\varphi\big)\delta + i\kappa\varphi\beta_1\big)\gamma$$
$$+ \big((\beta_2 a + \beta_1 i + \varepsilon v + \kappa)\sigma + \kappa(\beta_2 a + \beta_1 i)\big)\omega^2$$
$$+ \left(\left(2\left(\kappa + \frac{\varphi}{2} + \frac{\delta}{2}\right)a\beta_2 + (\beta_1 i + \varepsilon v + \kappa)\delta + (2\beta_1 i + \varphi)\kappa + (\beta_1 i + \varepsilon v)\varphi\right)\, \sigma\right.$$
$$+ \kappa(\varphi + \delta)(\beta_2 a + \beta_1 i)\big)\omega + (a((\kappa$$
$$+ \varphi)\delta + \kappa\varphi)\beta_2 + \big((\beta_1 i + \varphi)\kappa + (\beta_1 i + \varepsilon v)\varphi\big)\delta + i\kappa\varphi\beta_1\big)\sigma + \kappa\varphi\delta(\beta_2 a + \beta_1 i)$$

$$
\begin{aligned}
b_5 = 21\mu^5 &+ (15\beta_2 a + 15\beta_1 i + 15\varepsilon v + 15\delta + 15\eta + 30\gamma + 15\kappa + 30\omega + 15\sigma + 15\varphi)\mu^4 \\
&+ \big((20\gamma + 20\omega + 10\sigma + ((10p - 10)s + 10a)\beta_2 + 10\delta + 10\kappa + 10\varphi \\
&+ (-10ps + 10i)\beta_1 + 10\varepsilon v)\eta + 10\gamma^2 \\
&+ (20\beta_2 a + 20\beta_1 i + 20\varepsilon v + 20\delta + 20\kappa + 30\omega + 20\sigma + 10\varphi)\gamma + 10\omega^2 \\
&+ (20\beta_2 a + 20\beta_1 i + 20\varepsilon v + 10\delta + 20\kappa + 20\sigma + 10\varphi)\omega \\
&+ (10\beta_2 a + 10\beta_1 i + 10\varepsilon v + 10\delta + 10\kappa + 10\varphi)\sigma + 10a(\kappa + \varphi + \delta)\beta_2 \\
&+ (10\beta_1 i + 10\delta + 10\varphi)\kappa + (10\beta_1 i + 10\varepsilon v + 10\varphi)\delta + 10(\beta_1 i + \varepsilon v)\varphi)\mu^3 \\
&+ \big((6\gamma^2 + (18\omega + 12\sigma + ((6p - 6)s + 12a)\beta_2 + 12\kappa + 12\delta + 6\varphi + (-6ps \\
&+ 12i)\beta_1 + 12\varepsilon v)\gamma + 6\omega^2 \\
&+ (12\sigma + ((12p - 12)s + 12a)\beta_2 + 12\kappa + 6\delta + 6\varphi + (-6ps + 12i)\beta_1 \\
&+ 12\varepsilon v)\omega \\
&+ (((6p - 6)s + 6a)\beta_2 + 6\kappa + 6\delta + 6\varphi + (-6ps + 6i)\beta_1 + 6\varepsilon v)\sigma + (((6p \\
&- 6)s + 6a)\kappa + ((6p - 6)s + 6a)\delta + ((6p - 6)s + 6a)\varphi + 6\varepsilon sv(p - 1))\beta_2 \\
&+ (6\delta + 6\varphi + (-6ps + 6i)\beta_1)\kappa + (6\varphi + (-6ps + 6i)\beta_1 + 6\varepsilon v)\delta \\
&+ (6\beta_1 i + 6\varepsilon v)\varphi - 6\varepsilon\beta_1 psv)\eta + (6\beta_2 a + 6\beta_1 i + 6\varepsilon v + 6\delta + 6\kappa + 6\omega \\
&+ 6\sigma)\gamma^2 + (6\omega^2 + (18\beta_2 a + 18\beta_1 i + 18\varepsilon v + 6\delta + 18\kappa + 18\sigma + 6\varphi)\omega \\
&+ (12\beta_2 a + 12\beta_1 i + 12\varepsilon v + 12\delta + 12\kappa + 6\varphi)\sigma + 12a\left(\kappa + \frac{\varphi}{2} + \delta\right)\beta_2 \\
&+ (12\beta_1 i + 12\delta + 6\varphi)\kappa + (12\beta_1 i + 12\varepsilon v + 6\varphi)\delta + 6(\beta_1 i + \varepsilon v)\varphi)\gamma \\
&+ (6\beta_2 a + 6\beta_1 i + 6\varepsilon v + 6\kappa + 6\sigma)\omega^2 \\
&+ \Bigg((12\beta_2 a + 12\beta_1 i + 12\varepsilon v + 6\delta + 12\kappa + 6\varphi)\sigma + 12\left(\kappa + \frac{\varphi}{2} + \frac{\delta}{2}\right)a\beta_2 \\
&+ (12\beta_1 i + 6\delta + 6\varphi)\kappa + 6(\beta_1 i + \varepsilon v)(\varphi + \delta)\Bigg)\omega \\
&+ (6a(\kappa + \varphi + \delta)\beta_2 + (6\beta_1 i + 6\delta + 6\varphi)\kappa \\
&+ (6\beta_1 i + 6\varepsilon v + 6\varphi)\delta + 6(\beta_1 i + \varepsilon v)\varphi)\sigma + 6a((\varphi + \delta)\kappa + \delta\varphi)\beta_2 \\
&+ ((6\beta_1 i + 6\varphi)\delta + 6\varphi\beta_1 i)\kappa + 6\varphi\delta(\beta_1 i + \varepsilon v)\big)\mu^2 \\
&+ \big(((3\beta_2 a + 3\beta_1 i + 3\varepsilon v + 3\delta + 3\kappa + 3\omega + 3\sigma)\gamma^2 \\
&+ (3\omega^2 + (9\sigma + ((3p - 3)s + 9a)\beta_2 + 9\kappa + 3\delta + 3\varphi + (-3ps + 9i)\beta_1 \\
&+ 9\varepsilon v)\omega + (((3p - 3)s + 3a)\beta_2 + 6\kappa + 6\delta + 3\varphi + (-3ps + 3i)\beta_1 + 6\varepsilon v)\sigma \\
&+ (((3p - 3)s + 6a)\kappa + ((3p - 3)s + 6a)\delta + 3a\varphi + 3\varepsilon sv(p - 1))\beta_2 + (6\delta \\
&+ 3\varphi + (+3ps + 6i)\beta_1)\kappa + (3\varphi + (-3ps + 6i)\beta_1 + 6\varepsilon v)\delta \\
&+ (3\beta_1 i + 3\varepsilon v)\varphi - 3\varepsilon\beta_1 psv)\gamma \\
&+ (3\sigma + ((3p - 3)s + 3a)\beta_2 + 3\varepsilon v + 3\beta_1 i + 3\kappa)\omega^2 \\
&+ \big((((6p - 6)s + 6a)\beta_2 + 6\kappa + 3\delta + 3\varphi + (-3ps + 6i)\beta_1 + 6\varepsilon v)\sigma + (((6p \\
&- 6)s + 6a)\kappa + ((3p - 3)s + 3a)\delta + ((3p - 3)s + 3a)\varphi + 6\varepsilon sv(p - 1))\beta_2 \\
&+ (3\delta + 3\varphi \\
&+ (-3ps + 6i)\beta_1)\kappa + (3\beta_1 i + 3\varepsilon v)\delta + (3\beta_1 i + 3\varepsilon v)\varphi - 3\varepsilon\beta_1 psv)\omega \\
&+ ((((3p - 3)s + 3a)\kappa + ((3p - 3)s + 3a)\delta + ((3p - 3)s + 3a)\varphi \\
&+ 3\varepsilon sv(p - 1))\beta_2 \\
&+ (3\delta + 3\varphi + (-3ps + 3i)\beta_1)\kappa + (3\varphi + (-3ps + 3i)\beta_1 + 3\varepsilon v)\delta \\
&+ (3\beta_1 i + 3\varepsilon v)\varphi - 3\varepsilon\beta_1 psv)\sigma + (3(a + (p - 1)s)(\varphi + \delta)\kappa + (((3p - 3)s \\
&+ 3a)\varphi + 3\varepsilon sv(p - 1))\delta + 3\varphi\varepsilon sv(p - 1))\beta_2 \\
&+ \big((3\varphi + (-3ps + 3i)\beta_1)\delta + 3\varphi\beta_1 i)\kappa + 3\delta\big((\beta_1 i + \varepsilon v)\varphi - \varepsilon\beta_1 psv\big)\big)\eta \\
&+ \big((3\beta_2 a + 3\beta_1 i + 3\varepsilon v + 3\kappa + 3\sigma)\omega + (3\beta_2 a + 3\beta_1 i + 3\varepsilon v + 3\delta + 3\kappa)\sigma \\
&+ 3a(\kappa + \delta)\beta_2 + (3\beta_1 i + 3\delta)\kappa + 3\delta(\beta_1 i + \varepsilon v)\big)\gamma^2 + \big((3\beta_2 a + 3\beta_1 i + 3\varepsilon v \\
&+ 3\kappa + 3\sigma)\omega^2 + \big((9\beta_2 a + 9\beta_1 i + 9\varepsilon v + 3\delta + 9\kappa + 3\varphi)\sigma
\end{aligned}
$$

$$+ 9\left(\kappa + \frac{\varphi}{3} + \frac{\delta}{3}\right) a\beta_2 + (9\beta_1 i + 3\delta + 3\varphi)\kappa + 3(\beta_1 i + \varepsilon v)(\varphi + \delta))\omega$$

$$+ \Big(6a\left(\kappa + \frac{\varphi}{2} + \delta\right)\beta_2 + (6\beta_1 i + 6\delta + 3\varphi)\kappa + (6\beta_1 i + 6\varepsilon v + 3\varphi)\delta$$

$$+ 3(\beta_1 i + \varepsilon v)\varphi)\sigma + 3((\varphi + 2\delta)\kappa$$

$$+ \delta\varphi)a\beta_2 + \big((6\beta_1 i + 3\varphi)\delta + 3\varphi\beta_1 i\big)\kappa + 3\varphi\delta(\beta_1 i + \varepsilon v)\Big)\gamma$$

$$+ \big((3\beta_2 a + 3\beta_1 i + 3\varepsilon v + 3\kappa)\sigma + 3\kappa(\beta_2 a + \beta_1 i)\big)\omega^2$$

$$+ \Bigg(\Big(6\left(\kappa + \frac{\varphi}{2} + \frac{\delta}{2}\right) a\beta_2 + (6\beta_1 i + 3\delta + 3\varphi)\kappa + 3(\beta_1 i + \varepsilon v)(\varphi$$

$$+ \delta)\Big)\sigma + 3\kappa(\varphi + \delta)(\beta_2 a + \beta_1 i)\Bigg)\omega$$

$$+ \Big(3a((\varphi + \delta)\kappa + \delta\varphi)\beta_2 + \big((3\beta_1 i + 3\varphi)\delta + 3\varphi\beta_1 i)\kappa + 3\varphi\delta(\beta_1 i + \varepsilon v)\big)\sigma$$

$$+ 3\kappa\varphi\delta(\beta_2 a + \beta_1 i)\Big)\mu$$

$$+ \Big(\big((\beta_2 a + \beta_1 i + \varepsilon v + \kappa + \sigma)\omega + (\varepsilon v + \delta + \kappa)\sigma + a(\kappa + \delta)\beta_2 + (\beta_1 i + \delta)\kappa$$

$$+ \delta(\beta_1 i + \varepsilon v)\big)\gamma^2 + \big((\beta_2 a + \beta_1 i + \varepsilon v + \kappa + \sigma)\omega^2$$

$$+ \big((((p-1)s + a(p+1))\beta_2 + 3\kappa + \delta + \varphi + (-ps + (p+1)i)\beta_1 + 3\varepsilon v)\sigma$$

$$+ (((p-1)s + 3a)\kappa + a\delta + a\varphi + \varepsilon sv(p-1))\beta_2$$

$$+ (\delta + \varphi + (-ps + 3i)\beta_1)\kappa + \delta(\beta_1 i + \varepsilon v) + (\beta_1 i + \varepsilon v)\varphi - \varepsilon\beta_1 psv)\omega$$

$$+ (((a + (p-1)s)\kappa + (a + (p-1)s)\delta + a\varphi p + \varepsilon sv(p-1))\beta_2$$

$$+ (2\delta + \varphi + (-ps + i)\beta_1)\kappa + (\varphi + (-ps + i)\beta_1 + 2\varepsilon v)\delta$$

$$+ (ip\beta_1 + \varepsilon v)\varphi - \varepsilon\beta_1 psv)\sigma + (((2a + (p-1)s)\delta + a\varphi)\kappa + \delta(a\varphi + \varepsilon sv(p$$

$$- 1)))\beta_2 + \big((\varphi + (-ps + 2i)\beta_1)\delta + \varphi\beta_1 i\big)\kappa + \delta\big((\beta_1 i + \varepsilon v)\varphi - \varepsilon\beta_1 psv)\big)\gamma$$

$$+ \big(((a + (p-1)s)\beta_2 + \varepsilon v + \beta_1 i + \kappa)\sigma + ((a + (p-1)s)\kappa + \varepsilon sv(p-1))\beta_2$$

$$+ i\kappa\beta_1)\omega^2 + ((((a + (p-1)s)\delta + (2a + (2p-2)s)\kappa + (a + (p-1)s)\varphi$$

$$+ 2\varepsilon sv(p-1))\beta_2 + (\delta + \varphi + (-ps + 2i)\beta_1)\kappa + \delta(\beta_1 i + \varepsilon v)$$

$$+ (\beta_1 i + \varepsilon v)\varphi - \varepsilon\beta_1 psv)\sigma + (((a + (p-1)s)\kappa + \varepsilon sv(p-1))\beta_2 + i\kappa\beta_1)(\varphi$$

$$+ \delta))\omega + (((a + (p-1)s)(\varphi + \delta)\kappa - (((a - s)\varphi - \varepsilon sv)\delta - \varphi\varepsilon sv)(p$$

$$- 1))\beta_2 + \big((\varphi + (-ps + i)\beta_1)\delta + \varphi\beta_1 i\big)\kappa$$

$$+ \delta((-i(p-1)\beta_1 + \varepsilon v)\varphi - \varepsilon\beta_1 psv)\big)\sigma + \delta(((a + (p-1)s)\kappa$$

$$+ \varepsilon sv(p-1))\beta_2 + i\kappa\beta_1)\varphi)\eta + \Big(\big((\beta_2 a + \beta_1 i + \varepsilon v + \kappa)\sigma + \kappa(\beta_2 a + \beta_1 i)\big)\omega$$

$$+ \big(a(\kappa + \delta)\beta_2 + (\beta_1 i + \delta)\kappa + \delta(\beta_1 i + \varepsilon v)\big)\sigma + \kappa\delta(\beta_2 a + \beta_1 i)\Big)\gamma^2$$

$$+ \Big(\big((\beta_2 a + \beta_1 i + \varepsilon v + \kappa)\sigma + \kappa(\beta_2 a + \beta_1 i)\big)\omega^2$$

$$+ \Bigg(\Big(3\left(\kappa + \frac{\varphi}{3} + \frac{\delta}{3}\right) a\beta_2 + (3\beta_1 i + \delta + \varphi)\kappa + (\beta_1 i + \varepsilon v)(\varphi + \delta))\sigma + \kappa(\varphi$$

$$+ \delta)(\beta_2 a + \beta_1 i)\Big)\omega + (((\varphi$$

$$+ 2\delta)\kappa + \delta\varphi)a\beta_2 + \big((2\beta_1 i + \varphi)\delta + \varphi\beta_1 i\big)\kappa$$

$$+ \varphi\delta(\beta_1 i + \varepsilon v)\Big)\sigma + \kappa\varphi\delta(\beta_2 a + \beta_1 i)\Bigg)\gamma + \kappa\sigma(\omega + \delta)(\omega + \varphi)(\beta_2 a + \beta_1 i)$$

$$
\begin{aligned}
b_6 = 7\mu^6\& &+ (6\beta_2 a + 6\beta_1 i + 6\varepsilon v + 6\delta + 6\eta + 12\gamma + 6\kappa + 12\omega + 6\sigma + 6\varphi)\mu^5 \\
&+ \bigl((10\gamma + 10\omega + (5a + (5p - 5)s)\beta_2 + 5\sigma + 5\delta + 5\varphi + 5\kappa + (-5ps \\
&+ 5i)\beta_1 + 5\varepsilon v)\eta + 5\gamma^2 + (10\beta_2 a + 10\beta_1 i + 10\varepsilon v + 10\delta + 10\kappa + 15\omega \\
&+ 10\sigma + 5\varphi)\gamma + (5\beta_2 a + 5\beta_1 i + 5\varepsilon v + 5\delta + 5\kappa + 10\omega + 5\varphi)\sigma + 5\omega^2 \\
&+ (10\beta_2 a + 10\beta_1 i + 10\varepsilon v + 5\delta + 10\kappa + 5\varphi)\omega + 5a(\kappa + \varphi + \delta)\beta_2 \\
&+ (5\beta_1 i + 5\delta + 5\varphi)\kappa + (5\beta_1 i + 5\varepsilon v + 5\varphi)\delta + 5(\beta_1 i + \varepsilon v)\varphi)\mu^4 \\
&+ \bigl((4\gamma^2 + (12\omega + 8\sigma + ((4p - 4)s + 8a)\beta_2 + 8\delta + 8\kappa + 4\varphi + (-4ps \\
&+ 8i)\beta_1 + 8\varepsilon v)\gamma + (8\omega \\
&+ ((4p - 4)s + 4a)\beta_2 + 4\kappa + 4\delta + 4\varphi + (-4ps + 4i)\beta_1 + 4\varepsilon v)\sigma + 4\omega^2 \\
&+ (((8p - 8)s + 8a)\beta_2 + 8\kappa + 4\delta + 4\varphi + (-4ps + 8i)\beta_1 + 8\varepsilon v)\omega + (((4p \\
&- 4)s + 4a)\delta + ((4p - 4)s + 4a)\kappa + ((4p - \&4)s + 4a)\varphi + 4\varepsilon sv(p \\
&- 1))\beta_2 + (4\delta + 4\varphi + (-4ps + 4i)\beta_1)\kappa \\
&+ (4\varphi + (-4ps + 4i)\beta_1 + 4\varepsilon v)\delta + (4\beta_1 i + 4\varepsilon v)\varphi - 4\varepsilon\beta_1 psv)\eta \\
&+ (4\beta_2 a + 4\beta_1 i + 4\varepsilon v + 4\delta + 4\kappa + 4\omega + 4\sigma)\gamma^2 \\
&+ \bigl((8\beta_2 a + 8\beta_1 i + 8\varepsilon v + 8\delta + 8\kappa + 12\omega + 4\varphi)\sigma + 4\omega^2 + (12\beta_2 a + 12\beta_1 i \\
&+ 12\varepsilon v + 4\delta + 12\kappa + 4\varphi)\omega + 8a\left(\kappa + \frac{\varphi}{2} + \delta\right)\beta_2 \\
&+ (8\beta_1 i + 8\delta + 4\varphi)\kappa + (8\beta_1 i + 8\varepsilon v + 4\varphi)\delta + 4(\beta_1 i + \varepsilon v)\varphi)\gamma + (4\omega^2 \\
&+ (8\beta_2 a + 8\beta_1 i + 8\varepsilon v + 4\delta + 8\kappa + 4\varphi)\omega + 4a(\kappa + \varphi \\
&+ \delta)\beta_2 + (4\beta_1 i + 4\delta + 4\varphi)\kappa + (4\beta_1 i + 4\varepsilon v + 4\varphi)\delta + 4(\beta_1 i + \varepsilon v)\varphi)\sigma \\
&+ (4\beta_2 a + 4\beta_1 i + 4\varepsilon v + 4\kappa)\omega^2 \\
&+ \Bigl(8\left(\kappa + \frac{\varphi}{2} + \frac{\delta}{2}\right)a\beta_2 + (8\beta_1 i + 4\delta + 4\varphi)\kappa + 4(\beta_1 i + \varepsilon v)(\varphi + \delta))\omega \\
&+ 4a((\varphi + \delta)\kappa + \delta\varphi)\beta_2 + \bigl((4\beta_1 i + 4\varphi)\delta + 4\varphi\beta_1 i\bigr)\kappa + 4\varphi\delta(\beta_1 i + \varepsilon v)\Bigr)\mu^3 \\
&+ \Bigl(\bigl((3\beta_2 a + 3\beta_1 i + 3\varepsilon v + 3\delta + 3\kappa + 3\omega + 3\sigma)\gamma^2 + ((9\omega + ((3p \\
&- 3)s + 3a)\beta_2 + 6\kappa + 6\delta + 3\varphi + (-3ps + 3i)\beta_1 + 6\varepsilon v)\sigma + 3\omega^2 \\
&+ (((3p - 3)s + 9a)\beta_2 + 9\kappa + 3\delta + 3\varphi + (-3ps + 9i)\beta_1 + 9\varepsilon v)\omega + (((3p \\
&- 3)s + 6a)\kappa + ((3p - 3)s + 6a)\delta + 3a\varphi + 3\varepsilon sv(p - 1))\beta_2 \\
&+ (6\delta + 3\varphi + (-3ps + 6i)\beta_1)\kappa + (3\varphi \\
&+ (-3ps + 6i)\beta_1 + 6\varepsilon v)\delta + (3\beta_1 i + 3\varepsilon v)\varphi - 3\varepsilon\beta_1 psv)\gamma + (3\omega^2 \\
&+ (((6p - 6)s + 6a)\beta_2 + 6\kappa + 3\delta + 3\varphi + (-3ps + 6i)\beta_1 + 6\varepsilon v)\omega + (((3p \\
&- 3)s + 3a)\kappa + ((3p - 3)s + 3a)\delta + ((3p - 3)s + 3a)\varphi + 3\varepsilon sv(p - 1))\beta_2 \\
&+ (3\delta + 3\varphi + (-3ps + 3i)\beta_1)\kappa + (3\varphi + (-3ps + 3i)\beta_1 + 3\varepsilon v)\delta \\
&+ (3\beta_1 i + 3\varepsilon v)\varphi - 3\varepsilon\beta_1 psv)\sigma \\
&+ (((3p - 3)s + 3a)\beta_2 + 3\varepsilon v + 3\beta_1 i + 3\kappa)\omega^2 + ((((6p - 6)s + 6a)\kappa \\
&+ ((3p - 3)s + 3a)\delta + ((3p - 3)s + 3a)\varphi + 6\varepsilon sv(p - 1))\beta_2 \\
&+ (3\delta + 3\varphi + (-3ps + 6i)\beta_1)\kappa \\
&+ (3\beta_1 i + 3\varepsilon v)\delta + (3\beta_1 i + 3\varepsilon v)\varphi - 3\varepsilon\beta_1 psv)\omega + (3(a + (p - 1)s)(\varphi \\
&+ \delta)\kappa + (((3p - 3)s + 3a)\varphi + 3\varepsilon sv(p - 1))\delta + 3\varphi\varepsilon sv(p - 1))\beta_2 \\
&+ \bigl((3\varphi + (-3ps + 3i)\beta_1)\delta + 3\varphi\beta_1 i\bigr)\kappa + 3\delta\bigl((\beta_1 i + \varepsilon v)\varphi - \varepsilon\beta_1 psv\bigr)\bigr)\eta \\
&+ \bigl((3\beta_2 a + 3\beta_1 i + 3\varepsilon v + 3\delta + 3\kappa + 3\omega)\sigma + (3\beta_2 a + 3\beta_1 i + 3\varepsilon v + 3\kappa)\omega \\
&+ 3a(\kappa + \delta)\beta_2 + (3\beta_1 i + 3\delta)\kappa + 3\delta(\beta_1 i + \varepsilon v)\bigr)\gamma^2 \\
&+ \bigl((3\omega^2 + (9\beta_2 a + 9\beta_1 i + 9\varepsilon v + 3\delta + 9\kappa + 3\varphi)\omega + 6a\left(\kappa + \frac{\varphi}{2} + \delta\right)\beta_2 \\
&+ (6\beta_1 i + 6\delta + 3\varphi)\kappa + (6\beta_1 i + 6\varepsilon v + 3\varphi)\delta + 3(\beta_1 i + \varepsilon v)\varphi)\sigma \\
&+ (3\beta_2 a + 3\beta_1 i + 3\varepsilon v + 3\kappa)\omega^2 \\
&+ \Bigl(9\left(\kappa + \frac{\varphi}{3} + \frac{\delta}{3}\right)a\beta_2 + (9\beta_1 i + 3\delta + 3\varphi)\kappa + 3(\beta_1 i + \varepsilon v)(\varphi + \delta))\omega
\end{aligned}
$$

$$+ 3((\varphi + 2\delta)\kappa + \delta\varphi)a\beta_2 + \big((6\beta_1 i + 3\varphi)\delta + 3\varphi\beta_1 i)\kappa + 3\varphi\delta(\beta_1 i + \varepsilon v)\big)\gamma$$
$$+ \big((3\beta_2 a + 3\beta_1 i + 3\varepsilon v + 3\kappa)\omega^2$$
$$+ \left(6\left(\kappa + \frac{\varphi}{2} + \frac{\delta}{2}\right) a\beta_2 + (6\beta_1 i + 3\delta + 3\varphi)\kappa + 3(\beta_1 i + \varepsilon v)(\varphi + \delta)\right)\omega$$
$$+ 3a((\varphi + \delta)\kappa + \delta\varphi)\beta_2$$
$$+ \big((3\beta_1 i + 3\varphi)\delta + 3\varphi\beta_1 i\big)\kappa + 3\varphi\delta(\beta_1 i + \varepsilon v)\big)\sigma + 3\kappa(\omega + \delta)(\omega$$
$$+ \varphi)(\beta_2 a + \beta_1 i)\Big)\mu^2$$
$$+ \Big(\big(((2\varepsilon v + 2\delta + 2\kappa + 2\omega)\sigma + (2\beta_2 a + 2\beta_1 i + 2\varepsilon v + 2\kappa)\omega + 2a(\kappa + \delta)\beta_2$$
$$+ (2\beta_1 i + 2\delta)\kappa + 2\delta(\beta_1 i + \varepsilon v)\big)\gamma^2 + ((2\omega)^2$$
$$+ (((2p - 2)s + 2a(p + 1))\beta_2 + 6\kappa + 2\delta + 2\varphi + (-2ps + (2p+2)i)\beta_1$$
$$+ 6\varepsilon v)\omega + ((2a + (2p - 2)s)\kappa + (2a + (2p - 2)s)\delta + 2a\varphi p + 2\varepsilon sv(p$$
$$- 1))\beta_2 + (4\delta + 2\varphi + (-2ps + 2i)\beta_1)\kappa + (2\varphi + (-2ps + 2i)\beta_1 + 4\varepsilon v)\delta$$
$$+ (2ip\beta_1 + 2\varepsilon v)\varphi - 2\varepsilon\beta_1 psv)\sigma + (2\beta_2 a + 2\beta_1 i + 2\varepsilon v + 2\kappa)\omega^2 + ((((2p$$
$$- 2)s + 6a)\kappa + 2a\delta + 2a\varphi + 2\varepsilon sv(p - 1))\beta_2 + (2\delta + 2\varphi$$
$$+ (-2ps + 6i)\beta_1)\kappa + (2\beta_1 i + 2\varepsilon v)\delta + (2\beta_1 i + 2\varepsilon v)\varphi - 2\varepsilon\beta_1 psv)\omega$$
$$+ ((((2p - 2)s + 4a)\delta + 2a\varphi)\kappa + 2\delta(a\varphi + \varepsilon sv(p - 1)))\beta_2$$
$$+ \big((2\varphi + (-2ps + 4i)\beta_1)\delta + 2\varphi\beta_1 i\big)\kappa + 2\delta\big((\beta_1 i + \varepsilon v)\varphi - \varepsilon\beta_1 psv)\big)\gamma$$
$$+ \big(((2a + (2p - 2)s)\beta_2 + 2\varepsilon v + 2\beta_1 i + 2\kappa)\omega^2 + ((((4p - 4)s + 4a)\kappa$$
$$+ (2a + (2p - 2)s)\delta + (2a + (2p - 2)s)\varphi + 4\varepsilon sv(p - 1))\beta_2$$
$$+ (2\delta + 2\varphi + (-2ps + 4i)\beta_1)\kappa + (2\beta_1 i + 2\varepsilon v)\delta + (2\beta_1 i + 2\varepsilon v)\varphi$$
$$- 2\varepsilon\beta_1 psv)\omega + (2(a + (p - 1)s)(\varphi + \delta)\kappa - 2(((a - s)\varphi - \varepsilon sv)\delta$$
$$- \varphi\varepsilon sv)(p - 1))\beta_2 + \big((2\varphi + (-2ps + 2i)\beta_1)\delta + 2\varphi\beta_1 i\big)\kappa$$
$$+ 2\delta\big((-i(p - 1)\beta_1 + \varepsilon v)\varphi - \varepsilon\beta_1 psv)\big)\sigma + 2(\omega + \delta)(((a + (p - 1)s)\kappa$$
$$+ \varepsilon sv(p - 1))\beta_2 + i\kappa\beta_1)(\omega + \varphi))\eta$$
$$+ \Big(\big((2\beta_2 a + 2\beta_1 i + 2\varepsilon v + 2\kappa)\omega + 2a(\kappa + \delta)\beta_2 + (2\beta_1 i + 2\delta)\kappa$$
$$+ 2\delta(\beta_1 i + \varepsilon v)\big)\sigma + 2\kappa(\omega + \delta)(\beta_2 a + \beta_1 i)\Big)\gamma^2$$
$$+ \Big(\big((2\beta_2 a + 2\beta_1 i + 2\varepsilon v + 2\kappa)\omega^2 + (6\left(\kappa + \frac{\varphi}{3} + \frac{\delta}{3}\right) a\beta_2$$
$$+ (6\beta_1 i + 2\delta + 2\varphi)\kappa + 2(\beta_1 i + \varepsilon v)(\varphi + \delta)\Big)\omega + 2((\varphi + 2\delta)\kappa + \delta\varphi)a\beta_2$$
$$+ \big((4\beta_1 i + 2\varphi)\delta + 2\varphi\beta_1 i\big)\kappa + 2\varphi\delta(\beta_1 i + \varepsilon v)\big)\sigma + 2\kappa(\omega + \delta)(\omega$$
$$+ \varphi)(\beta_2 a + \beta_1 i)\Big)\gamma + 2\kappa\sigma(\omega + \delta)(\omega + \varphi)(\beta_2 a + \beta_1 i)\big)\mu + ((\omega + \delta)((\varepsilon v$$
$$+ \kappa)\sigma + \kappa(\beta_2 a + \beta_1 i))\gamma^2 + \Big(\big((ap\beta_2 + ip\beta_1 + \varepsilon v + \kappa)\omega^2 + ((((p - 1)s$$
$$+ a(p + 1))\kappa + ap\delta + a\varphi p + \varepsilon sv(p - 1))\beta_2 + (\delta + \varphi$$
$$+ (-ps + (p + 1)i)\beta_1)\kappa + (ip\beta_1 + \varepsilon v)\delta + (ip\beta_1 + \varepsilon v)\varphi - \varepsilon\beta_1 psv)\omega$$
$$+ (((a + (p - 1)s)\delta + a\varphi p)\kappa + \varepsilon sv\delta(p - 1))\beta_2 + ((\varphi$$
$$+ (-ps + i)\beta_1)\delta + \varphi\beta_1 ip)\kappa + \varepsilon v\delta(-ps\beta_1 + \varphi)\big)\sigma + \kappa(\omega$$
$$+ \delta)(\omega + \varphi)(\beta_2 a + \beta_1 i)\Big)\gamma$$
$$+ \sigma\big((((a + (p - 1)s)\kappa + \varepsilon sv(p - 1))\beta_2 + i\kappa\beta_1)\omega^2$$
$$+ (((a + (p - 1)s)\kappa + \varepsilon sv(p - 1))\beta_2$$
$$+ i\kappa\beta_1)(\varphi + \delta)\omega - \delta(p - 1)(((a - s)\kappa - \varepsilon sv)\beta_2 + i\kappa\beta_1)\varphi)\big)\eta + \kappa\sigma\gamma(\omega$$
$$+ \delta)(\omega + \varphi + \gamma)(\beta_2 a + \beta_1 i)$$

$$
\begin{aligned}
b_7 = \mu^7 &+ (a\beta_2 + \beta_1 i + \varepsilon v + \delta + \eta + 2\gamma + \kappa + 2\omega + \sigma + \varphi)\mu^6 + ((2\gamma \\
&+ \sigma + 2\omega + (a + (p-1)s)\beta_2 + \kappa + \delta + \varphi + (-ps + i)\beta_1 + \varepsilon v)\eta + \gamma^2 \\
&+ (2a\beta_2 + 2\beta_1 i + 2\varepsilon v + 2\delta + 2\kappa + 3\omega + 2\sigma + \varphi)\gamma + (a\beta_2 + \beta_1 i + \varepsilon v + \delta \\
&+ \kappa + 2\omega + \varphi)\sigma + \omega^2 + (2a\beta_2 + 2\beta_1 i + 2\varepsilon v + \delta + 2\kappa + \varphi)\omega + a(\kappa + \varphi \\
&+ \delta)\beta_2 + (\beta_1 i + \delta + \varphi)\kappa + (\beta_1 i + \varepsilon v + \varphi)\delta + (\beta_1 i + \varepsilon v)\varphi)\mu^5 \\
&+ \big((\gamma^2 + (3\omega + (2a + (p-1)s)\beta_2 + 2\sigma + 2\delta + \varphi + 2\kappa + (-ps + 2i)\beta_1 \\
&+ 2\varepsilon v)\gamma + (2\omega + (a + (p-1)s)\beta_2 + \kappa + \delta + \varphi + (-ps + i)\beta_1 + \varepsilon v)\sigma + \omega^2 \\
&+ ((2a + (2p-2)s)\beta_2 + \delta + \varphi + 2\kappa + (-ps + 2i)\beta_1 + 2\varepsilon v)\omega + ((a + (p \\
&- 1)s)\delta + (a + (p-1)s)\varphi + (a + (p-1)s)\kappa + \varepsilon s v(p-1))\beta_2 + (\delta + \varphi \\
&+ (-ps + i)\beta_1)\kappa + (\varphi + (-ps + i)\beta_1 + \varepsilon v)\delta + (\beta_1 i + \varepsilon v)\varphi - \varepsilon\beta_1 psv)\eta \\
&+ (a\beta_2 + \beta_1 i + \varepsilon v + \delta + \kappa + \omega + \sigma)\gamma^2 + \big((2a\beta_2 + 2\beta_1 i + 2\varepsilon v + 2\delta + 2\kappa \\
&+ 3\omega + \varphi)\sigma + \omega^2 + (3a\beta_2 + 3\beta_1 i + 3\varepsilon v + \delta + 3\kappa + \varphi)\omega \\
&+ 2a\Big(\kappa + \frac{\varphi}{2} + \delta\Big)\beta_2 + (2\beta_1 i + 2\delta + \varphi)\kappa \\
&+ (2\beta_1 i + 2\varepsilon v + \varphi)\delta + (\beta_1 i + \varepsilon v)\varphi)\gamma \\
&+ (\omega^2 + (2a\beta_2 + 2\beta_1 i + 2\varepsilon v + \delta + 2\kappa + \varphi)\omega + a(\kappa + \varphi + \delta)\beta_2 \\
&+ (\beta_1 i + \delta + \varphi)\kappa + (\beta_1 i + \varepsilon v + \varphi)\delta + (\beta_1 i + \varepsilon v)\varphi)\sigma \\
&+ (a\beta_2 + \beta_1 i + \varepsilon v + \kappa)\omega^2 \\
&+ \Bigg(2\Big(\kappa + \frac{\varphi}{2} + \frac{\delta}{2}\Big)a\beta_2 + (2\beta_1 i + \delta + \varphi)\kappa + (\beta_1 i + \varepsilon v)(\varphi + \delta)\Bigg)\omega \\
&+ a((\varphi + \delta)\kappa + \delta\varphi)\beta_2 + \big((\beta_1 i + \varphi)\delta + \varphi\beta_1 i\big)\kappa + \varphi\delta(\beta_1 i + \varepsilon v)\Bigg)\mu^4 \\
&+ \Bigg(\Big((a\beta_2 + \beta_1 i + \varepsilon v + \delta + \kappa + \omega + \sigma)\gamma^2 + ((3\omega + (a + (p-1)s)\beta_2 + 2\kappa \\
&+ 2\delta + \varphi + (-ps + i)\beta_1 + 2\varepsilon v)\sigma + \omega^2 \\
&+ (((p-1)s + 3a)\beta_2 + 3\kappa + \delta + \varphi + (-ps + 3i)\beta_1 + 3\varepsilon v)\omega + ((2a + (p \\
&- 1)s)\delta + (2a + (p-1)s)\kappa + a\varphi + \varepsilon s v(p-1))\beta_2 \\
&+ (2\delta + \varphi + (-ps + 2i)\beta_1)\kappa + (\varphi + (-ps + 2i)\beta_1 + 2\varepsilon v)\delta \\
&+ (\beta_1 i + \varepsilon v)\varphi - \varepsilon\beta_1 psv)\gamma \\
&+ (\omega^2 + ((2a + (2p-2)s)\beta_2 + \delta + \varphi + 2\kappa + (-ps + 2i)\beta_1 + 2\varepsilon v)\omega + ((a \\
&+ (p-1)s)\delta + (a + (p-1)s)\varphi + (a + (p-1)s)\kappa + \varepsilon s v(p-1))\beta_2 \\
&+ (\delta + \varphi + (-ps + i)\beta_1)\kappa + (\varphi + (-ps + i)\beta_1 + \varepsilon v)\delta + (\beta_1 i + \varepsilon v)\varphi \\
&- \varepsilon\beta_1 psv)\sigma + ((a + (p-1)s)\beta_2 + \varepsilon v + \beta_1 i + \kappa)\omega^2 + (((a + (p-1)s)\delta \\
&+ (2a + (2p-2)s)\kappa + (a + (p-1)s)\varphi + 2\varepsilon s v(p-1))\beta_2 + (\delta \\
&+ \varphi + (-ps + 2i)\beta_1)\kappa + \delta(\beta_1 i + \varepsilon v) + (\beta_1 i + \varepsilon v)\varphi - \varepsilon\beta_1 psv)\omega + ((a + (p \\
&- 1)s)(\varphi + \delta)\kappa + ((a + (p-1)s)\varphi + \varepsilon s v(p-1))\delta + \varphi\varepsilon s v(p-1))\beta_2 \\
&+ \big((\varphi + (-ps + i)\beta_1)\delta + \varphi\beta_1 i\big)\kappa + \delta\big((\beta_1 i + \varepsilon v)\varphi - \varepsilon\beta_1 psv\big)\big)\eta \\
&+ \big((a\beta_2 + \beta_1 i + \varepsilon v + \delta + \kappa + \omega)\sigma + (a\beta_2 + \beta_1 i + \varepsilon v + \kappa)\omega + a(\kappa + \delta)\beta_2 \\
&+ (\beta_1 i + \delta)\kappa + \delta(\beta_1 i + \varepsilon v)\big)\gamma^2 \\
&+ \Bigg(\Bigg(\omega^2 + (3a\beta_2 + 3\beta_1 i + 3\varepsilon v + \delta + 3\kappa + \varphi)\omega + 2a\Big(\kappa + \frac{\varphi}{2} + \delta)\beta_2 \\
&+ (2\beta_1 i + 2\delta + \varphi)\kappa + (2\beta_1 i + 2\varepsilon v + \varphi)\delta + (\beta_1 i + \varepsilon v)\varphi)\sigma \\
&+ (a\beta_2 + \beta_1 i + \varepsilon v + \kappa)\omega^2 \\
&+ \Bigg(3\Big(\kappa + \frac{\varphi}{3} + \frac{\delta}{3}\Big)a\beta_2 + (3\beta_1 i + \delta + \varphi)\kappa + (\beta_1 i + \varepsilon v)(\varphi + \delta))\omega + ((\varphi \\
&+ 2\delta)\kappa + \delta\varphi)a\beta_2 + \big((2\beta_1 i + \varphi)\delta + \varphi\beta_1 i)\kappa + \varphi\delta(\beta_1 i + \varepsilon v)\big)\gamma \\
&+ \big((a\beta_2 + \beta_1 i + \varepsilon v + \kappa)\omega^2 \\
&+ \Bigg(2\Big(\kappa + \frac{\varphi}{2} + \frac{\delta}{2}\Big)a\beta_2 + (2\beta_1 i + \delta + \varphi)\kappa + (\beta_1 i + \varepsilon v)(\varphi + \delta))\omega + a((\varphi \\
&+ \delta)\kappa + \delta\varphi)\beta_2
\end{aligned}
$$

$$+\Big((\beta_1 i+\varphi)\delta+\varphi\beta_1 i\Big)\kappa+\varphi\delta(\beta_1 i+\varepsilon v)\Big)\sigma+\kappa(\omega+\delta)(\omega$$
$$+\varphi)(a\beta_2+\beta_1 i)\Big)\mu^3+((((\varepsilon v+\delta+\kappa+\omega)\sigma+(a\beta_2+\beta_1 i+\varepsilon v+\kappa)\omega$$
$$+a(\kappa+\delta)\beta_2+(\beta_1 i+\delta)\kappa+\delta(\beta_1 i+\varepsilon v)\Big)\gamma^2$$
$$+\Big((\omega^2+(((p-1)s+a(p+1))\beta_2+3\kappa+\delta+\varphi+(-ps+(p+1)i)\beta_1$$
$$+3\varepsilon v)\omega+((a+(p-1)s)\kappa+(a+(p-1)s)\delta+a\varphi p+\varepsilon sv(p-1))\beta_2$$
$$+(2\delta+\varphi+(-ps+i)\beta_1)\kappa+(\varphi+(-ps+i)\beta_1+2\varepsilon v)\delta$$
$$+(ip\beta_1+\varepsilon v)\varphi-\varepsilon\beta_1 psv)\sigma+(a\beta_2+\beta_1 i+\varepsilon v+\kappa)\omega^2+((((p-1)s$$
$$+3a)\kappa+a\delta+a\varphi+\varepsilon sv(p-1))\beta_2+(\delta+\varphi+(-ps+3i)\beta_1)\kappa$$
$$+\delta(\beta_1 i+\varepsilon v)+(\beta_1 i+\varepsilon v)\varphi-\varepsilon\beta_1 psv)\omega+(((2a+(p-1)s)\delta+a\varphi)\kappa$$
$$+\delta(a\varphi+\varepsilon sv(p-1)))\beta_2$$
$$+\Big((\varphi+(-ps+2i)\beta_1)\delta+\varphi\beta_1 i\Big)\kappa+\delta\Big((\beta_1 i+\varepsilon v)\varphi-\varepsilon\beta_1 psv\Big)\Big)\gamma$$
$$+\Big(((a+(p-1)s)\beta_2+\varepsilon v+\beta_1 i+\kappa)\omega^2+(((a+(p-1)s)\delta+(2a+(2p$$
$$-2)s)\kappa+(a+(p-1)s)\varphi+2\varepsilon sv(p-1))\beta_2+(\delta+\varphi+(-ps+2i)\beta_1)\kappa$$
$$+\delta(\beta_1 i+\varepsilon v)+(\beta_1 i+\varepsilon v)\varphi-\varepsilon\beta_1 psv)\omega+((a+(p-1)s)(\varphi+\delta)\kappa-(((a$$
$$-s)\varphi-\varepsilon sv)\delta-\varphi\varepsilon sv)(p-1))\beta_2+((\varphi$$
$$+(-ps+i)\beta_1)\delta+\varphi\beta_1 i)\kappa+\delta\Big((-i(p-1)\beta_1+\varepsilon v)\varphi-\varepsilon\beta_1 psv\Big)\Big)\sigma+(\omega$$
$$+\delta)(((a+(p-1)s)\kappa+\varepsilon sv(p-1))\beta_2+i\kappa\beta_1)(\omega+\varphi))\eta$$
$$+\Bigg(\Big((a\beta_2+\beta_1 i+\varepsilon v+\kappa)\omega+a(\kappa+\delta)\beta_2+(\beta_1 i+\delta)\kappa+\delta(\beta_1 i+\varepsilon v))\sigma$$
$$+\kappa(\omega+\delta)(a\beta_2+\beta_1 i)\Big)\gamma^2+\Bigg(\Big((a\beta_2+\beta_1 i+\varepsilon v+\kappa)\omega^2$$
$$+\left(3\left(\kappa+\frac{\varphi}{3}+\frac{\delta}{3}\right)a\beta_2+(3\beta_1 i+\delta+\varphi)\kappa+(\beta_1 i+\varepsilon v)(\varphi+\delta)\right)\omega+((\varphi$$
$$+2\delta)\kappa+\delta\varphi)a\beta_2$$
$$+\Big((2\beta_1 i+\varphi)\delta+\varphi\beta_1 i\Big)\kappa+\varphi\delta(\beta_1 i+\varepsilon v)\Big)\sigma+\kappa(\omega+\delta)(\omega$$
$$+\varphi)(a\beta_2+\beta_1 i)\Big)\gamma+\kappa\sigma(\omega+\delta)(\omega+\varphi)(a\beta_2+\beta_1 i)\Big)\mu^2$$
$$+\Big(((\omega+\delta)((\varepsilon v+\kappa)\sigma+\kappa(a\beta_2+\beta_1 i)\Big)\gamma^2$$
$$+\Bigg(\Big((ap\beta_2+ip\beta_1+\varepsilon v+\kappa)\omega^2+((((p-1)s+a(p+1))\kappa+ap\delta+a\varphi p$$
$$+\varepsilon sv(p-1))\beta_2+(\delta+\varphi+(-ps$$
$$+(p+1)i)\beta_1)\kappa+(ip\beta_1+\varepsilon v)\delta+(ip\beta_1+\varepsilon v)\varphi-\varepsilon\beta_1 psv)\omega+(((a+(p$$
$$-1)s)\delta+a\varphi p)\kappa+\varepsilon sv\delta(p-1))\beta_2+((\varphi$$
$$+(-ps+i)\beta_1)\delta+\varphi\beta_1 ip)\kappa+\varepsilon v\delta(-ps\beta_1+\varphi)\Big)\sigma+\kappa(\omega$$
$$+\delta)(\omega+\varphi)(a\beta_2+\beta_1 i)\Big)\gamma$$
$$+\sigma\Big(((a+(p-1)s)\kappa+\varepsilon sv(p-1))\beta_2+i\kappa\beta_1)\omega^2$$
$$+(((a+(p-1)s)\kappa+\varepsilon sv(p-1))\beta_2$$
$$+i\kappa\beta_1)(\varphi+\delta)\omega-\delta(p-1)(((a-s)\kappa-\varepsilon sv)\beta_2+i\kappa\beta_1)\varphi)\Big)\eta$$
$$+\kappa\sigma\gamma(\omega+\delta)(\omega+\varphi+\gamma)(a\beta_2+\beta_1 i)\Big)\mu+\kappa\omega\sigma p\gamma(\omega+\varphi+\delta)(a\beta_2+\beta_1 i)\eta$$

$$
\begin{aligned}
B_1 = {} & ((a-(1-p)s)\beta_2 + (i-ps)\beta_1 + v\varepsilon + \kappa + 2\omega + \sigma + \varphi + 6\mu + \delta + 2\gamma)\eta^2 \\
& + (a(a-(1-p)s)\beta_2^2 + (((2i-ps)a - is(1-p))\beta_1 + (4a-(1-p)s)\gamma \\
& + (14a - 2(1-p)s)\mu + 2a(v\varepsilon+\delta+\kappa+2\omega+\sigma+\varphi))\beta_2 + i(i-ps)\beta_1^2 \\
& + ((4i-ps)\gamma + (14i-2ps)\mu + (4i-ps)\omega + (2i-ps)\varphi + 2i(v\varepsilon+\delta+\kappa \\
& + \sigma))\beta_1 + (v\varepsilon+\delta+2\gamma+\kappa+8\mu+2\omega+\sigma+\varphi)(v\varepsilon+\delta+2\gamma+\kappa+6\mu \\
& + 2\omega+\sigma+\varphi))\eta + a^2(\kappa+2\omega+\sigma+\varphi+6\mu+\delta+2\gamma)\beta_2^2 + (2i(\kappa+2\omega \\
& + \sigma+\varphi+6\mu+\delta+2\gamma)\beta_1 + 4\gamma^2 + (4v\varepsilon+4\delta+4\kappa+28\mu+8\omega+4\sigma \\
& + 4\varphi)\gamma + 48\mu^2 + (12v\varepsilon+14\delta+14\kappa+28\omega+14\sigma+14\varphi)\mu + 4\omega^2 + (4v\varepsilon \\
& + 4\delta+4\kappa+4\sigma+4\varphi)\omega + \varphi^2 + (2v\varepsilon+2\delta+2\kappa+2\sigma)\varphi + \delta^2 + (2v\varepsilon+2\kappa \\
& + 2\sigma)\delta + \sigma^2 + (2v\varepsilon+2\kappa)\sigma + \kappa(v\varepsilon+\kappa))a\beta_2 + i^2(\kappa+2\omega+\sigma+\varphi+6\mu \\
& + \delta+2\gamma)\beta_1^2 + (4\gamma^2 + (4v\varepsilon+4\delta+4\kappa+28\mu+8\omega+4\sigma+4\varphi)\gamma + 48\mu^2 \\
& + (12v\varepsilon+14\delta+14\kappa+28\omega+14\sigma+14\varphi)\mu + 4\omega^2 + (4v\varepsilon+4\delta+4\kappa \\
& + 4\sigma+4\varphi)\omega + \varphi^2 + (2v\varepsilon+2\delta+2\kappa+2\sigma)\varphi + \delta^2 + (2v\varepsilon+2\kappa+2\sigma)\delta \\
& + \sigma^2 + (2v\varepsilon+2\kappa)\sigma + \kappa(v\varepsilon+\kappa))i\beta_1 + 2\gamma^3 + (4v\varepsilon+4\delta+4\kappa+26\mu+7\omega \\
& + 4\sigma+3\varphi)\gamma^2 + (96\mu^2 + (28v\varepsilon+28\delta+28\kappa+54\omega+28\sigma+26\varphi)\mu \\
& + 7\omega^2 + (8v\varepsilon+8\delta+8\kappa+8\sigma+6\varphi)\omega + \varphi^2 \\
& + (4v\varepsilon+4\delta+4\kappa+4\sigma)\varphi + 2(v\varepsilon+\delta+\kappa+\sigma)^2)\gamma + 112\mu^3 + (48v\varepsilon+48\delta \\
& + 48\kappa+96\omega+48\sigma+48\varphi)\mu^2 + (26\omega^2 + (28v\varepsilon+26\delta+28\kappa+28\sigma \\
& + 26\varphi)\omega + 6\varphi^2 + (14v\varepsilon+14\delta+14\kappa+14\sigma)\varphi + 6\delta^2 + (14v\varepsilon+14\kappa \\
& + 14\sigma)\delta + 6\sigma^2 + (14v\varepsilon+14\kappa)\sigma + 6(v\varepsilon+\kappa)^2)\mu + 2\omega\omega^3 + (4v\varepsilon+3\delta+4\kappa \\
& + 4\sigma+3\varphi)\omega^2 \\
& + (\varphi^2 + (4v\varepsilon+4\delta+4\kappa+4\sigma)\varphi + \delta^2 + (4v\varepsilon+4\kappa+4\sigma)\delta + 2(v\varepsilon+\kappa \\
& + \sigma)^2)\omega + (v\varepsilon+\delta+\kappa+\sigma)\varphi^2 + (v\varepsilon+\delta+\kappa+\sigma)^2\varphi + (\sigma+\delta)(v\varepsilon+\delta \\
& + \kappa)(v\varepsilon+\kappa+\sigma)
\end{aligned}
$$
